# Quantum Reality in the Selective Reduction of a Benzofuran System

**DOI:** 10.3390/molecules24112061

**Published:** 2019-05-30

**Authors:** Arturo Coaviche-Yoval, Erik Andrade-Jorge, Cuauhtémoc Pérez-González, Héctor Luna, Ricardo Tovar-Miranda, José G. Trujillo-Ferrara

**Affiliations:** 1 Doctorado en Ciencias Biológicas y de la Salud, Universidad Autónoma Metropolitana-Unidad Xochimilco, Mexico City 04960, Mexico; a.cy2@hotmail.com; 2 Departamento de Bioquímica, Sección de Estudios de Posgrado e Investigación, Escuela Superior de Medicina, Instituto Politécnico Nacional, Mexico City 11340, Mexico; andrade136@hotmail.com; 3 Unidad de Investigación en Biomedicina, Facultad de Estudios Superiores-Iztacala, Universidad Nacional Autónoma de México. Av. de los Barrios 1, Los Reyes Iztacala, Tlalnepantla 54090, Estado de México, Mexico; 4 Departamento de Sistemas Biológicos, Universidad Autónoma Metropolitana-Unidad Xochimilco, Mexico City 04960, Mexico; cperezg@correo.xoc.uam.mx (C.P.-G.); lchm1964@correo.xoc.uam.mx (H.L.); 5 Instituto de Ciencias Básicas, Universidad Veracruzana, Xalapa 91190, Veracruz, Mexico

**Keywords:** catalytic reduction, olefinic carbons, selectivity, global reactivity, local reactivity, electrophilic

## Abstract

Two 2,3-disubstituted benzofurans (**1** and **2**), analogs of gamma-aminobutyric acid (GABA), were synthesized to obtain their 2,3-dihydro derivatives from the Pd/C-driven catalytic reduction of the double bond in the furanoid ring. The synthesis produced surprising by-products. Therefore, theoretical calculations of global and local reactivity were performed based on Pearson’s hard and soft acids and bases (HSAB) principle to understand the regioselectivity that occurred in the reduction of the olefinic carbons of the compounds. Local electrophilicity (**ω*_k_***) was the most useful parameter for explaining the selectivity of the polar reactions. This local parameter was defined with the condensed Fukui function and redefined with the electrophilic (***P_k_^+^***) Parr function. The similar patterns of both resulting sets of values helped to demonstrate the electrophilic behavior (soft acid) of the olefinic carbons in these compounds. The theoretical calculations, nuclear magnetic resonance, and resonance hybrids showed the moieties in each compound that are most susceptible to reduction.

## 1. Introduction

Compounds of the benzofuran series and the corresponding 2,3-dihydroderivatives are receiving considerable attention due to their biological activity and chemical properties. They are used to treat traumatic and ischemic central nervous system (CNS) lesions, arteriosclerosis, liver diseases, and cerebrovascular diseases [1,2,3]. They serve as the starting material for synthesising new compounds [4], such as the frequently-reported synthesis of 2,3-dihydrobenzofurans. One might think that the hydrogenation of the corresponding 2,3-disubstituted benzofuran would be the most direct route for its synthesis, since the reaction of hydrogen with the benzofuran is a simple addition to the double bond of the furanoid ring to produce 2,3-dihydrobenzofurans. However, hydrogenation is more difficult with oxygen-heterocycles than nitrogen-heterocycles and many other aromatic compounds. In the former case, the catalysts based on group VIII metals and their oxides promote hydrogenation of the heterocyclic ring, and can also stimulate the reaction to proceed with future saturation of the benzene ring, producing the octahydrobenzofuran derivatives. After hydrogenolytic cleavage of the furanoid ring to produce phenols and alcohols, future reactions may lead to the elimination of oxygen with the formation of alkyl-benzenes and cyclohexanes. 

**Scheme 1:**
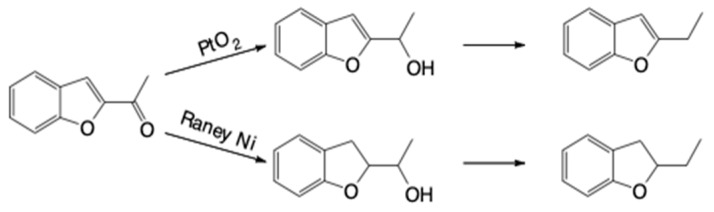
Catalytic reduction with platinum oxide and Raney nickel.

Raney nickel is the most active catalyst in the hydrogenation of benzofuran to 2,3-dihydrobenzofuran. With platinum oxide, the hydrogenation of the benzene ring occurs at a considerably lower rate compared to that of the double bond of the heterocyclic ring. In the presence of Pd/C, the rate of hydrogen absorption at 25 °C is considerably lower for benzofuran than indene or furan. In the hydrogenation of 2-acetylbenzofuran, platinum oxide displays high selectivity, only reducing the acetyl group, whereas Raney nickel also hydrogenates the C=C double bond of the heterocycle (Scheme 1) [4,5,6,7].

Given advances in computational chemistry, enormous progress has been made in the characterization of different structural and electronic properties of atoms and molecules. Consequently, it is now possible to predict reactivity and reaction mechanisms, design better synthesis strategies, and explain unexpected results in organic synthesis [8,9] using density functional theory (DFT), which is an important theoretical tool in chemistry and physics [10,11].

To more reliably predict reaction outcomes in organic synthesis, many parameters have been described in computational chemistry, such as global and local reactivity based on Pearson’s acid-base principle. This hard-soft concept has been used extensively to interpret reactivity and selectivity. The global parameters encompass global hardness (**η**), global softness (***S***), chemical potential (***µ***), the electrophilicity (**ω**) and nucleophilicity (**N**) indices, as well as electron-donating power (**ω^−^**) and electron-accepting power (**ω^+^**). The local parameters include the Fukui functions ***f***(***k***), the Parr functions ***P***(***r***), the local electrophilicity (**ω*_k_***) and nucleophilicity (**N*_k_***) indices, and the local reactivity difference index (***R_k_***). The two Fukui functions are ***f*_k_^+^** (for atom ***k*** as an electrophile that is susceptible to receiving an attack from a nucleophile) and ***f*_k_^−^** (for atom ***k*** as a nucleophile and able to perform an attack against an electrophile). The two Parr functions are ***P*_k_^+^** (the local Parr function for nucleophilic attacks) and ***P_k_*^−^** (the local Parr function for electrophilic susceptibility) [12,13,14,15,16,17,18,19].

**Scheme 2:**
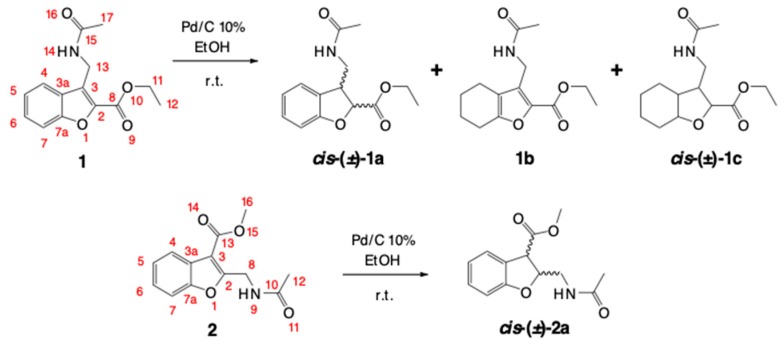
Products of the catalytic hydrogenation of benzofurans **1** and **2**.

The present study describes the synthesis of two isomers of 2,3-disubstituted benzofurans (**1** and **2**), analogs of GABA, and the subsequent catalytic hydrogenation with Pd/C to obtain their *cis*-2,3-dihydroderivatives. The application of 10% Pd/C for the selective reduction of the α,β-unsaturated C=C double bond in the furanoid ring generated surprising hydrogenated products (Scheme 2).

Our aim was to seek the theoretical-experimental coherence that could account for the outcome of the catalytic reduction (with 10% Pd/C) of the C=C double bond of the furanoid ring in ethyl 3- (acetamidomethyl)benzofuran-2-carboxylate (**1**) and methyl 2-(acetamidomethyl)benzofuran-3- carboxylate (**2**) (Scheme 2). For this purpose, descriptors of global and local reactivity were calculated, based on the DFT framework.

## 2. Results and Discussion 

### 2.1. Chemistry 

#### 2.1.1. General Methodology for the Synthesis of **1**

The synthesis of compound **1** was previously reported by our group [20].

**Scheme 3:**
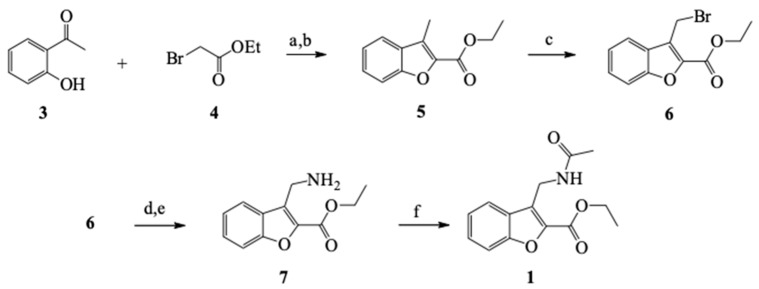
General synthetic pathway to obtain compound **1**. Reaction conditions: (a) Dimethylformamide (DMF), K_2_CO_3_, 125 °C; (b) EtOH, H_2_SO_4_, 40 °C; (c) CCl_4_, *N*-bromosuccinimide (NBS), benzoyl peroxide, reflux; (d) CHCl_3_, C_6_H_12_N_4_, reflux; (e) H_2_O, reflux; and (f) Dichloromethane (DCM), Et_3_N, Ac_2_O, CH_3_COCl, room temperature (rt).

A Williamson-type etherification between 2-acetylphenol **3** and ethyl bromoacetate **4** was followed by an intramolecular aldol condensation to furnish the benzofuran **5** core. Subsequently, benzylic bromination was completed by free radicals and the bromine was replaced by the amino group through an S_N_2 reaction with hexamethylenetetramine. The amino group was acetylated in **7** to produce **1**, which had a global yield of 23% (Scheme 3). The infrared (IR) spectrum shows a band of N–H at 3,291 cm^−1^, the C=O of the ester at 1,712 cm^−1^, and the carbonyl of the amide at 1,653 cm^−1^. In the ^1^H-nuclear magnetic resonance (NMR) spectrum (with CDCl_3_), there is a four-signal system from 7.88 to 7.30 ppm for aromatic protons, a broad signal at 6.36 for the NH proton, a double signal at 4.85 corresponding to the benzylic protons of methylene alpha of the amino group, an A_2 × 3_ system for the ethyl fragment of the ester (*q* 4.48 ppm, *t* 1.45 ppm), and a singlet at 1.96 ppm characteristic of the acetyl group. The peak in high-resolution mass spectrometry (HRMS) M^+^ + 1 (*m/z*) at 261.105 confirms the structure of **1** (Supplementary Materials).

#### 2.1.2. General Methodology for the Synthesis of **2**

**Scheme 4:**
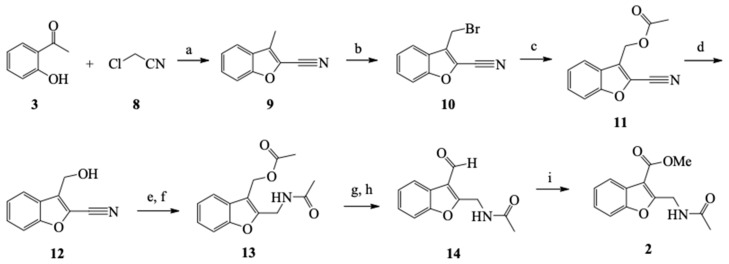
General synthetic pathway for preparing compound **2**. Reaction conditions: (a) DMF, K_2_CO_3_, 140 °C; (b) CCl_4_, NBS, benzoyl peroxide, reflux; (c) AcOH, AcOK, reflux; (d) MeOH, H_2_SO_4_, rt; (e) MeOH, NaBH_4_-CoCl_4_·6H_2_O, rt; (f) DCM, Et_3_N, CH_3_COCl, rt; (g) MeOH, K_2_CO_3_, rt; (h) DCM, PCC, AcONa, rt; and (i) MeOH, KCN, NaCN, AcOH, MnO_2_, rt.

Etherification of the corresponding phenol **3** with chloroacetonitrile and subsequent intramolecular aldol condensation produced benzofuran **9** [21]. The methyl group was functionalized by benzylic bromination, followed by treatment of the brominated product with potassium acetate to produce **11** and then hydrolysis of the acetate group to generate **12** [22]. Afterward, the reduction of the nitrile group furnished the amino-alcohol, which became acetylated to produce the diacetylated **13** [23,24,25,26]. The acetate group of **13** was selectively hydrolyzed with potassium carbonate to deprotect the hydroxyl group and form the oxidized product (aldehyde) **14** [27,28]. The synthesis of **2** was completed by the oxidation of **14** with manganese oxide in the presence of cyanide to form the methyl ester (5.4% overall yield for the synthetic pathway, Scheme 4) [29,30,31]. 

The IR spectrum showed a stretch band of N–H at 3,289 cm^−1^, a band at 1,713 cm^−1^ indicating the presence of the carbonyl carbon (C=O) of the ester, and a band at 1655 cm^−1^ confirming the existence of the carbonyl carbon (C=O) of the amide. The proton NMR (^1^H-NMR) spectrum (CDCl_3_) presents four aromatic protons between δ 7.95 and 7.32 ppm, a wide signal at 6.44 ppm for the NH proton, a doublet at 4.90 ppm that integrates the methylene CH_2_NHCO-, a singlet at 3.97 ppm for the methyl ester, and finally a simple signal at 2.02 ppm that integrates for the amide acetyl NHCOCH_3_. The HRMS peak M^+^ + 1 (*m/z*) at 248.08 confirms the structure of **2** (Supplementary Materials). 

#### 2.1.3. General Methodology for the Hydrogenation Reactions 

All hydrogenation reactions proceeded in glass tubes sealed with rubber stoppers equipped with needles [32,33,34]. The tubes were placed inside a flask, and the reaction was performed at room temperature (rt) in a parr hydrogenation device at 60 psi H_2_ pressure and with a 10% Pd/C catalyst. The reaction crudes corresponding to each hydrogenation experiment were analyzed by ^1^H-NMR and the relative proportions of each compound were calculated from the integral of the signal corresponding to the methyls of the acetyl group (NHCOCH_3_). The reaction mixtures were purified by flash silica column chromatography with hexane-ethyl acetate as the eluent. The isolated products were characterised by their NMR spectroscopic data, identifying the chemical shifts (δ, ppm) of the typical signals of **1** and its hydrogenated products (**1a**–**1c**), as well as of **2** and its hydrogenated derivative ***rac***-**2a** (Table 1; Supplementary Materials). 

The relative magnitude of the coupling constants (*J* = Hz) in the series of 2,3-dihydrobenzofurans is *J*_cis-2,3_ > *J*_tras-2,3_, in agreement with the Karplus equation. The NMR evidence showed that the relative stereochemistry of **1a**, **1c**, and **2a** is *cis*. The coupling constant for H2-H3 in the reduced products (*J*_H2-H3_ = 9.1 Hz in **1a**, *J*_H2-H3_ = 10.2 Hz and *J*_H3a-H7a_ = 8.4 Hz in **1c**, and *J*_H2-H3_ = 7.2 Hz in **2a**) corresponds to the expected values for the *cis* isomers [32,35,36] (Supplementary Materials).

**Figure 1:**
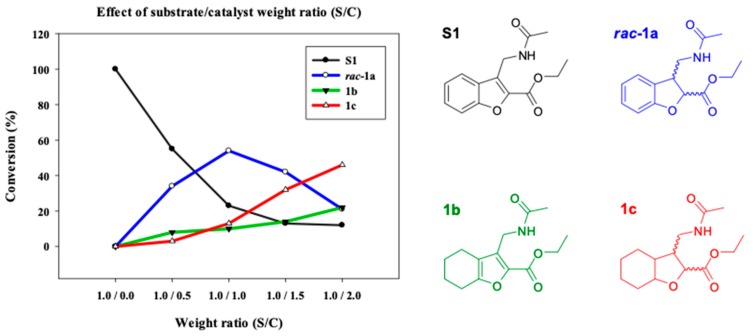
Variation in the hydrogenated products obtained (%) from compound **1** at 24 h due to the effect of the S/C ratio (entries 1–4, Table 2).

To optimize the reduction of the α,β-unsaturated C=C double bond of the furanoid ring in compounds **1** and **2**, experiments were conducted with different concentrations of the catalysts and distinct reaction times (Table 2). In the beginning, the attempts to perform the hydrogenation of **1** by applying minimum amounts of catalyst were unsuccessful (Table 2). The results demonstrate the possibility of reducing the α,β-unsaturated C=C double bond in the furanoid ring to furnish the target compound ***rac*-1a**. Curiously, the reaction generated a by-product of the partial reduction of the benzene ring **1b**. When the amount of catalyst increased, the reaction led to the total reduction of the heterocycle to produce the octahydrobenzofuran derivative **1c** (Figure 1). 

The preparation of ***rac*-1a** was optimal when using an S:C ratio of 1:1 (Figure 1, graphs from entries 1–4; Table 2). A higher proportion of catalyst caused a significant decrease in the target compound ***rac*-1a** and a significant increase in the proportion of the completely reduced compound **1c**. Therefore, **1c** appears to be mainly a by-product of ***rac*-1a**. 

**Figure 2:**
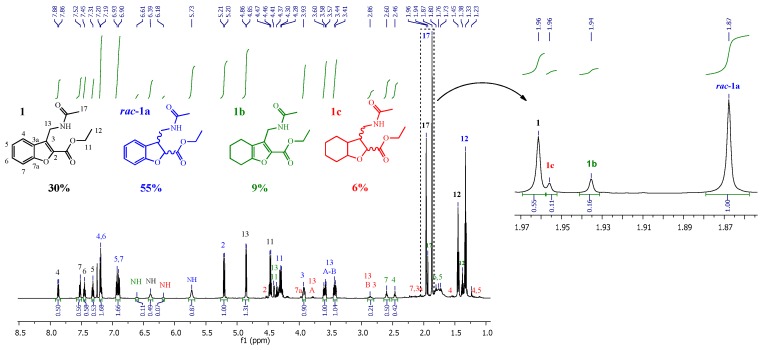
^1^H-NMR spectrum (CDCl_3_) of the crude reaction (entry 6, Table 2).

**Figure 3:**
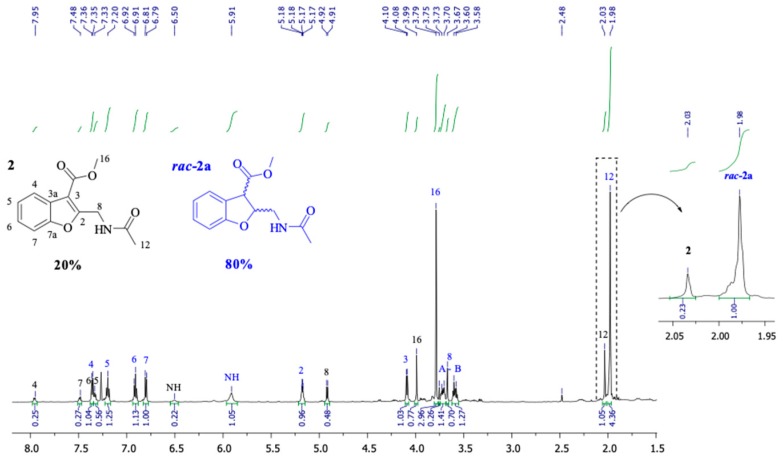
^1^H-NMR spectrum (CDCl_3_) of the crude reaction (entry 12, Table 2).

Unlike the case of **1**, the hydrogenation of the α,β-unsaturated C=C double bond in the furanoid ring of **2** was fairly simple and selective, producing ***rac*-2a** as the main product. The relative proportions of each product were calculated by analyzing the ^1^H-NMR spectral data of the reaction mixture from each experiment, taking advantage of the difference in the chemical shifts of the methyl protons of the acetyl group (CH_3_-17 and CH_3_-12) in the distinct compounds resulting from the reaction (Table 1, and Figure 2 and Figure 3).

### 2.2. Computational Studies (Theoretical Calculations)

#### 2.2.1. Conformational and Optimization Geometry

The optimized geometries of the structures of **1** and **2** are shown in Figures S3–S5 (Supplementary Materials).

#### 2.2.2. Indices of Global and of Local Reactivity 

Theoretical calculations were used to explore possible reasons for the susceptibility to reduction of the α,β-unsaturated C=C double bond of the furanoid ring in **1** and **2**, and for the unexpectedly yielded reduction by-products. Hence, we determined the global and local reactivity [37] (Table 3), which allowed for analysis of the selectivity of the reduction pathway (Table 4).

The potential ionization (***I***) and electron affinity (***A***) express the ability of a chemical species to donate and accept an electron, respectively. The chemical potentials of **1** and **2** were −4.0833 and −4.0301 eV, respectively, suggesting that **1** is more electron accepting and **2** is more electron donating. The most stable systems are those with the highest hardness values (those having the greatest variation between the Highest Occupied Molecular Orbital and Lowest Unoccupied Molecular Orbital (HOMO-LUMO). Since softness is the reciprocal of hardness, the most reactive systems possess greater softness. The present global softness values were not capable of indicating the most reactive system, evidenced by the lack of significant difference between **1** and **2** (***S*** = 0.1267 and 0.1261 eV, respectively). 

The electrophilicity index **ω** expresses the stabilization energy of an electrophile when it is saturated by electrons from the external environment. It also serves to characterize nucleophilicity, as a low electrophilicity value corresponds to elevated nucleophilicity. Based on the results, both compounds can be classified as moderate electrophiles. Thus, the data point to a greater electrophilic behavior for **1** than **2** (**ω** = 1.056 versus 1.025 eV, respectively). This tentative conclusion was confirmed by the nucleophilicity index **N**, which showed a greater nucleophilicity for **2** than **1** (**N** = 1.125 versus 1.087, respectively). This nucleophilicity index refers to tetracyanoethylene (TCE, ***I*** = 9.118 eV). The electron donating power (**ω^−^**) and electron accepting power (**ω^+^**) have a behavior similar to ***I*** and ***A***. The capacity of a chemical system to donate or accept a small fraction of charge is expressed as **ω^−^** and **ω^+^**, where **ω^−^** refers to the propensity of the system to donate electron density and **ω+** refers to its propensity to accept electron density. 

All the above information provides insights into global reactivity but not selectivity. The reactivity of a particular site of a molecular species can be explained through the FF (border function *f*(r)), which is a measure of the variation of the chemical potential in relation to the external potential at a particular point (r) of the molecule. The FF varies in accordance with the variation in the electron density ***ρ***(**r**) from point to point in a molecule due to the extraction of electrons from HOMO or addition of these to LUMO. 

The electrophilic and nucleophilic local softness (**s*_k_*^+^** and **s*_k_*^−^**) are given by FF multiplied by the global softness ***S***. Similar to the **s_k_**, other local descriptors are the indices of local electrophilicity **ω*_k_*** and nucleophilicity **N*_k_***, where **ω*_k_*** = **ω*f_k_*^+^** and **N*_k_*** = **N*f_k_*^−^**. They allow for the characterization of the most electrophilic and nucleophilic centers in an organic molecule by revealing the distribution of the indices of global electrophilicity **ω** and nucleophilicity **N** at the atomic sites ***k***. In a detailed analysis of FF, however, Yang-Mortier (YM) presented some relevant errors. Even though the local functions are normalized, the sum of the YM FF (***f_k_*^+^** or ***f_k_*^−^**) corresponding to heavy atoms (CH, CH_2_ and CH_3_) is below 1.0, a discrepancy with the sum of the Parr functions (***P_k_*^+^** or ***P_k_*^−^**), which is closer to 1.0. Using the Parr functions, the local indices for electrophilicity **ω*_k_*** and nucleophilicity **N*_k_*** were redefined. The relevant function for the reduction of olefinic carbons in **1** and **2** is local electrophilicity **ω*_k_***. A large value of this parameter at a particular site denotes greater reactivity toward a nucleophile. To analyze the Fukui functions and compare them to the proposed Parr functions, the corresponding local electrophilic values **ω_k_** are given in Table 4. 

**Figure 4:**
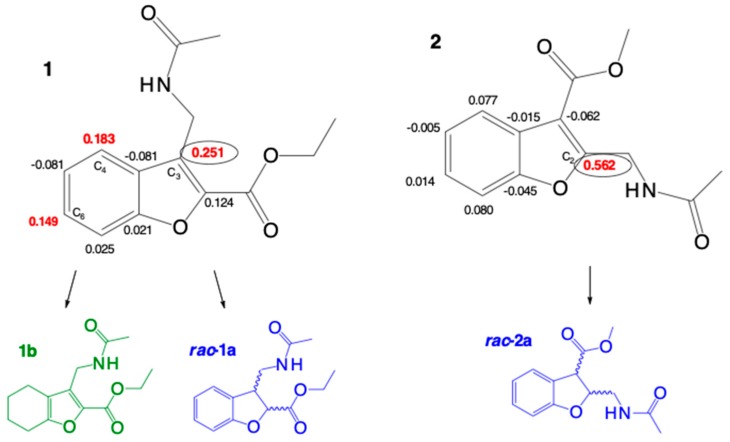
Indices of local electrophilicity ***ω_k_*** (Parr) in compounds **1** and **2**.

A preliminary examination of the data revealed similar patterns in both models for the local reactivity of molecules **1** and **2**. As shown, **1** contains three olefinic carbons, the most electrophilically activated centers (C3, C4, and C6), indicating its susceptibility to nucleophilic attack and to the consequent reduction by a polarized hydrogen induced by the catalyst. In **2**, conversely, only one electrophilically activated center (C2) is susceptible to reduction (Figure 4).

**Scheme 5:**
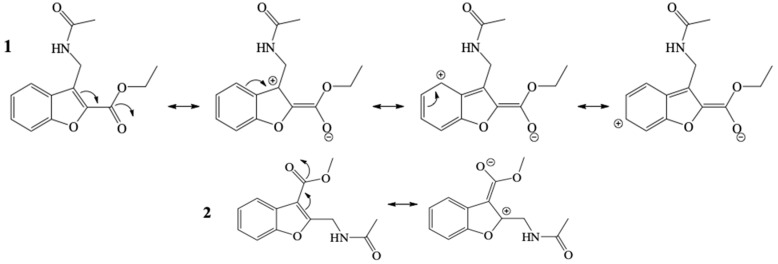
Resonance structures for compounds **1** and **2.**

α,β-unsaturated carbonyl compounds present the most electrophilic center at the β conjugated position, and electrophilicity is expressed well by the Parr and the Fukui functions. This parameter is in clear agreement with the regioselectivity experimentally observed in the catalytic reduction of **1** and **2**, as well as with the zwitterionic species of the resonance structures (Scheme 5). 

The α,β-unsaturated C=C double bond in **1** extends the conjugation of the entire heterocyclic system from the carbonyl carbon of the ester to the benzene ring, making it more aromatic and more electrophilic than **2**. The sites having greater electrophilicity, as observed in the resonance structures (Scheme 4), coincide with the values of local softness **s*_k_*^+^** and **ω*_k_***. In addition, the chemical shifts of the ^13^C-NMR spectra of the olefinic carbons are in agreement with the electron density ***ρ***(**r**) of these atoms, indicating their electrophilicity. The δ of the olefinic carbons centers that show relatively high electrophilicity for **1** (δ_C_) are 122 (C4), 127 (C3), and 128 (C6). The corresponding carbon center for **2** (δ_C_) is 162 (C2) (Supplementary Materials: Figures S2 and S20, respectively). Thus, these values denote a greater susceptibility of **1** to receiving a local nucleophilic attack. The experimental results of catalytic reduction are corroborated by the theoretical calculations of global and local reactivity.

## 3. Materials and Methods 

### 3.1. General Information 

Reagents and solvents were purchased from Sigma-Aldrich (Toluca, Edo. De Mexico, Mexico). Thin layer chromatography (TLC) was conducted on Merck 60 F_254_ silica gel plates (Toluca, Edo. De Mexico, Mexico) eluted with mixtures of hexane-AcOEt and read with ultraviolet (UV) light at 254 nm. The products were purified on a chromatography column with Merck (Toluca, Edo. De Mexico, Mexico) silica gel 60 (0.040–0.063 mm), and the corresponding structures were assigned by ^1^H and ^13^C-NMR on an Agilent-600 apparatus (at 600 MHz and 150 MHz, respectively, Mexico City, Mexico) with CDCl_3_ and CD_3_OD as solvent and tetramethylsilane (TMS) as the internal reference. Chemical shifts (δ) are reported in ppm and coupling constants (*J*) in hertz (Hz). Each signal is described as a singlet (s), doublet (d), triplet (t), quadruplet (q), multiplet (m), or broad signal (bs). Melting points were determined on a FisherScientific apparatus (Thermo Fisher Scientific, Waltham, MA, USA) and are uncorrected. Fourier transform infrared (FT-IR) spectra were recorded on a Perkin-Elmer Spectrum BX-II spectrophotometer (Mexico City, Mexico). The high performance liquid chromatography (HPLC) analysis (Agilent 1260; Agilent Technologies, Mexico City, Mexico) was carried out on a Chiralcel OJ-H column (4.6 × 250 mm; Chiral technologies Europe, bd girthier d’andernach, Illkirch, France), using a hexane:i-PrOH (80:20) mixture as the mobile phase at a flow rate of 0.6 mL/min and at 25 °C, and with a UV detector at a wavelength of 279 nm. Electrospray ionization high-resolution mass spectrometry (ESI-HRMS) was performed on a Bruker micrOTOF-Q-II apparatus (0.4 bar at rt, setting the dry heater at 180 °C, Mexico City, Mexico). The UHPLC 1290 Infinity II was coupled to electrospray ionization Quadrupole Time-Of-Flight (QTOF) 6545 (Agilent Technologies, Mexico City, Mexico).

### 3.2. Chemistry

*Ethyl 3-(acetamidomethyl)benzofuran-2-carboxylate* (**1**) [20]. Yellow solid (23% yield): (Ratio of front (Rf) *=* 0.36, Hex/AcOEt 4:6); m.p. 136–137 °C; IR (cm^−1^): 3291, 1712, 1653, 1598; ^1^H-NMR (600 MHz; CDCl_3_): δ 7.87 (1H, d, *J* = 8.4 Hz, CH-4), 7.52 (1H, d, *J* = 7.8 Hz, CH-7), 7.45 (1H, t, *J* = 8.4 Hz, CH-6), 7.32 (1H, t, *J* = 7.2 Hz, CH-5), 6.36 (1H, bs, NH), 4.86 (2H, d*, J* = 6.2 Hz, CH_2_-13), 4.47 (2H, q*, J* = 7.2 Hz, CH_2_-11), 1.96 (3H, s, CH_3_-17), 1.45 (3H, t, *J* = 7.2 Hz, CH_3_-12); ^13^C-NMR (150 MHz; CDCl_3_): δ 169.6 (C-15), 160.2 (C-8), 154.3 (C-7a), 141.8 (C-2), 128.3 (CH-6), 127.1 (C-3a), 126.9 (C-3), 123.8 (CH-5), 122.0 (CH-4), 112.1 (CH-7), 61.7 (CH_2_-11), 32.4 (CH_2_-13), 23.2 (CH_3_-17), 14.3 (CH_3_-12); HRMS (ESI^+^): calculated for C_14_H_15_NO_4_Na [M + Na]^+^, 284.090; found 284.087; HPLC on a Chiralcel OJ-H column (4.6 × 250 mm; Chiral technologies Europe, bd girthier d’andernach, Illkirch, France), with the mobile phase of an 80:20 Hex:*i*-PrOH system at a flow rate of 0.6 mL/min and at 25 °C, and with the UV light detector at λ = 279 nm. Retention time (*R*t) = 11.12 min. 

*Ethyl (±)-3-(acetamidomethyl)-2,3-dihydrobenzofuran-2-carboxylate* (***rac*-1a**) [20]. White crystals: (Rf = 0.18, Hex/AcOEt 4:6); m.p. 117–118 °C; IR (cm^−1^): 3311, 2936, 1750, 1638, 1597; ^1^H-NMR (600 MHz; CDCl_3_): δ 7.22–7.17 (2H, m, CH-4, CH-6), 6.95-6.88 (2H, m, CH-5, CH-7), 5.70 (1H, bs, NH), 5.21 (1H, d, *J* = 9.1 Hz, CH-2), 4.30 (2H, qq, *J* = 10.8, 7.2 Hz, CH_2_-11); 3.96–3.90 (1H, m, CH-3), 3.60 (1H*,* ddd, *J* = 14.1, 6.5, 4.9 Hz, CH_2_-13A), 3.43 (1H*,* ddd, *J* = 14.1, 6.3, 5.6 Hz, CH_2_-13B), 1.87 (3H, s, CH_3_-17), 1.34 (3H, t, *J* = 7.2 Hz, CH_3_-12); ^13^C-NMR (150 MHz; CDCl_3_): δ 172.8 (C-8), 172.5 (C-15), 161.5 (C-7a), 132.1 (CH-6), 128.8 (C-3a), 127.3 (CH-4), 124.4 (CH-5), 112.9 (CH-7), 84.8 (CH-2), 64.7 (CH_2_-11), 47.3 (CH-3), 42.6 (CH_2_-13), 25.9 (CH_3_-17), 16.9 (CH_3_-12); HRMS (ESI^+^): calculated for C_14_H_17_NO_4_Na [M + Na]^+^, 286.110; found 286.105; [α]${}_{D}^{20}$ = –0.002; The racemic mixture was examined by HPLC on a Chiralcel OJ-H column (4.6 × 250 mm; Chiral technologies Europe, bd girthier d’andernach, Illkirch, France), with the mobile phase of an 80:20 Hex:i-PrOH system at a flow rate of 0.6 mL/min and at 25 °C, and with the UV light detector at λ = 279 nm. *R*t_1_ = 13.10, *R*t_2_ = 18.83 min.

*Ethyl 3-(acetamidomethyl)-4,5,6,7-tetrahydrobenzofuran-2-carboxylate* (**1b**) [20]. Beige crystals: (Rf = 0.29, Hex/AcOEt 4:6); m.p. 115–116 °C; IR (cm^−1^): 3282, 2937, 1698, 1657; ^1^H-NMR (600 MHz; CDCl_3_): δ 6.61 (1H, bs, NH), 4.41 (2H, d, *J* = 6.1 Hz, CH_2_-13), 4.36 (2H, q, *J* = 7.2 Hz, CH_2_-11), 2.60 (2H, ddd, *J* = 6.3, 3.9, 1.5 Hz, CH_2_-7), 2.47 (2H, tt, *J* = 6.0, 1.5 Hz, CH_2_-4), 1.94 (3H, s, CH_3_-17), 1.81 (2H, m, CH_2_-6), 1.72 (2H, m, CH_2_-5), 1.38 (3H, t, *J* = 7.2 Hz, CH_3_-12); ^13^C-NMR (150 MHz; CDCl_3_): δ 172.3 (C-15), 162.5 (C-8), 158.0 (C-7a), 141.6 (C-2), 135.1 (C-3), 123.1 (C-3a), 63.6 (CH_2_-11), 35.5 (CH_2_-13), 26.2 (CH_2_-7), 25.9 (CH_3_-17), 25.1 (CH_2_-6), 25.0 (CH_2_-5), 22.9 (CH_2_-4), 17.1 (CH_3_-12); HRMS (ESI^+^): calculated for C_14_H_19_NO_4_Na [M + Na]^+^, 288.120; found 288.117; HPLC on a Chiracel OJ-H column (4.6 × 250 mm; Chiral technologies Europe, bd girthier d’andernach, Illkirch, France), with the mobile phase of an 80:20 Hex:*i*-PrOH system at a flow rate of 0.6 mL/min and at 25 °C, and with the UV light detector at λ = 279 nm. *R*t = 8.83 min. 

*Ethyl 3-(acetamidomethyl)octahydrobenzofuran-2-carboxylate* (**1c**). White oil: (Rf = 0.12 Hex/AcOEt 3:7); IR (cm^−1^): 3299, 2935, 1745, 1651, 1188; ^1^H-NMR (600 MHz; CDCl_3_): δ 6.28 (1H, bs, NH), 4.55 (1H, d, *J* = 10.0 Hz, CH-2), 4.36–4.18 (2H, m, CH_2_-11), 4.00 (1H, q, *J* = 8.4, 6, 3.1 Hz, CH-7a), 3.77 (1H, m, CH_2_-13A), 2.93 (1H, m, CH_2_-13B), 2.87 (1H, m, CH-3), 2.11 (1H, m, CH_2_-7), 2.02 (1H, m, CH-3a), 1.98 (3H, s, CH_3_-16), 1.74–1.68 (1H, m, CH_2_-5), 1.62–1.52 (2H, m, CH_2_-6, CH_2_-7), 1.52–1.47 (1H, m, CH_2_-6), 1.44–1.38 (1H, m, CH_2_-4), 1.34 (3H, t, CH_3_-12), 1.27–1.19 (1H, m, CH_2_-4), 1.16–1.10 (1H, m, CH_2_-5); ^13^C-NMR (150 MHz; CDCl_3_): δ 172.9 (C-8), 170.0 (C-15), 78.4 (CH-7a), 77.1 (CH-2), 61.2 (CH_2_-11), 48.1 (CH-3), 39.5 (CH-3a), 36.7 (CH_2_-13), 28.1 (CH_2_-7), 24.3 (CH_2_-5), 23.4 (CH_3_-17), 22.2 (CH_2_-4), 20.0 (CH_2_-6), 14.1 (CH_3_-12); HRMS (ESI^+^): calculated for C_14_H_23_NO_4_, 269.16; found 270.1721 (M + 1). 

*3-methylbenzofuran-2-carbonitrile* (**9**) [21]. To a mixture of 2′-hydroxyacetophenone (**3**) (6.80 g, 50 mmol) and K_2_CO_3_ (48.37 g, 350 mmol) in 65 mL of DMF without drying, chloroacetonitrile (**8**) (6.96 mL, 110 mmol) was added. The solution was stirred at rt under nitrogen atmosphere for 18 h. It was subsequently heated at 140 °C for 3 h. After cooling to rt, 150 mL of water was added and extracted with Et_2_O (3 × 200 mL), and the ether mixture was washed with water (3 × 250 mL) and brine (1 × 250 mL). The organic phase was dried with Na_2_SO_4_ and evaporated to produce a dark brown solid, which was purified on a SiO_2_ column (hexane/AcOEt, 95:5) to deliver **9** as beige crystals (5.98 g, 76%): (Rf = 0.7, hexane/AcOEt, 85:15); m.p. 105 °C; IR (cm^−1^): 2926, 2220, 1445, 1247, 1102; ^1^H-NMR (600 MHz; CDCl_3_): δ 7.61 (1H, dt, *J* = 7.8, 1.2 Hz, CH-4), 7.50 (1H, d, *J* = 0.9 Hz, CH-7), 7.49 (1H, t, *J* = 1.2 Hz, CH-6), 7.38–7.32 (1H, m, CH-5), 2.46 (3H, s, CH_3_-10); ^13^C-NMR (150 MHz; CDCl_3_): δ: 155.4 (C-7a), 129.7 (C-3), 128.4 (CH-6), 126.9 (C-3a), 124.9 (C-2), 123.9 (CH-5), 120.9 (CH-4), 112.1 (CH-7), 111.8 (C-8), 8.7 (CH_3_-10). 

*(2-cyanobenzofuran-3-yl)methyl acetate* (**11**) [20,21,22]. To benzofuran **9** (1 g, 6.37 mmol) in 18 mL of CCl_4_ we added *N*-bromosuccinimide (2.267 g, 12.74 mmol) and benzoyl peroxide (20 mg, 0.08 mmol). The reaction mixture was heated (under constant stirring) to reflux for 8 h under nitrogen atmosphere. After cooling to rt, it was filtered and the filtrate was evaporated to produce **10** as an amber solid (Rf = 0.65, hexane/AcOEt 85:15). Following dissolution of the reaction crude (without purification) in 30 mL AcOH, AcOK (3.12 g, 31.85 mmol) was added and heated to reflux for 1 h. Once the mixture was cooled, 20 mL of water was added and extracted with DCM (2 × 50 mL), and then the organic phase was washed with a saturated solution of NaHCO_3_ (2 × 80 mL), water (1 × 80 mL), and brine (1 × 80 mL). The mixture was dried with Na_2_SO_4_, filtered, and evaporated, resulting in a brown oily substance that was purified on a column of flash silica gel with hexane/AcOEt 90:10 to produce 930 mg (68%) of **11** as beige crystals: (Rf = 0.45, hexane/AcOEt 85:15); m.p. 38–39 °C; IR (cm^−1^): 2926, 2231, 1746, 1600, 1447, 1368, 1187, 1028; ^1^H-NMR (600 MHz; CDCl_3_): δ 7.68 (1H, dt, *J* = 7.8, 1.2 Hz, CH-4), 7.52 (1H, d, *J* = 1.2 Hz, CH-7), 7.51 (1H, t, *J* = 1.2 Hz, CH-6), 7.37 (1H, ddd, *J* = 8.4, 4.5, 3.6 Hz, CH-5), 5.38 (2H, s, CH_2_-10), 2.15 (3H, s, CH_3_-13); ^13^C-NMR (150 MHz; CDCl_3_): δ 170.4 (C-12), 155.5 (C-7a), 128.8 (CH-6), 127.9 (C-3a), 125.9 (C-3), 124.8 (C-2), 124.6 (CH-5), 121.3 (CH-4), 112.2 (CH-7), 111.0 (C-8), 55.8 (CH_2_-10), 20.5 (CH_3_-13). 

*3-(hidroxymethyl)benzofuran-2-carbonitrile* (**12**). A solution of **11** (500 mg, 2.33 mmol) in 10 mL of absolute MeOH and 0.5 mL of concentrated H_2_SO_4_ was heated at reflux for 4 h. After cooling, 20 mL of water was added and extracted with DCM (2 × 25 mL), and then the organic phase was washed with water (2 × 40 mL) and brine (1 × 40 mL). The organic extract was treated with Na_2_SO_4_ before being filtered and evaporated to dryness to complete the synthesis of **12**, yielding 400 mg (99%) as beige crystals: (Rf = 0.43, hexane/AcOEt 75:25); m.p. 85–87 °C; IR (cm^–1^): 3409, 2925, 2230, 1447, 1186, 1016; ^1^H-NMR (600 MHz; CDCl_3_): δ 7.77 (1H, dt, *J* = 7.8, 1.2 Hz, CH-4), 7.52–7.49 (2H, m, CH-7, CH-6), 7.36 (1H, ddd, *J* = 8.0, 5.2, 2.9 Hz, CH-5), 4.99 (2H, s, CH_2_-10); ^13^C-NMR (150 MHz; CDCl_3_): δ 155.7 (C-7a), 132.4 (C-3a), 128.7 (CH-6), 125.1 (C-3), 124.6 (C-2), 124.4 (CH-5), 121.7 (CH-4), 112.2 (CH-7), 111.4 (C-8), 55.5 (CH_2_-10). 

*(2-(acetamidomethyl)benzofuran-3-yl)methyl acetate* (**13**) [23,24,25,26]. To a mixture of **12** (400 mg, 2.31 mmol) and cobalt chloride (550 mg, 2.31 mmol) in 10 mL of MeOH (HPLC grade) at rt, sodium borohydride was carefully added in small portions (193 mg, 5.10 mmol). The solution was stirred under nitrogen atmosphere for 30 min, and then the reaction was completed by carefully adding 10 mL of water and 1 mL of 5N HCl. Subsequently, the mixture was made alkaline (pH ≈ 9) by adding concentrated NH_4_OH, filtered under vacuum through celite, and rinsed with 10 mL water. The filtrate was treated with DCM (3 × 20 mL), the organic phase was washed with brine (1 × 40 mL) and dried with Na_2_SO_4_, and the solvent was evaporated to produce 240 mg of the residue. Without purification, the amino alcohol crude was dissolved with 15 mL of DCM before adding 0.56 mL (4.06 mmol) of triethylamine and stirring under nitrogen atmosphere. Little by little, 0.3 mL (4.06 mmol) of acetyl chloride was added and the mixture was left at rt for 12 h (under constant stirring). To the reaction crude, 20 mL of DCM was added and the mixture was washed with water (2 × 25 mL) and brine (1 × 25 mL), then dried with Na_2_SO_4_, filtered, and concentrated to dryness. The crude was purified on a rotor with hexane/AcOEt (40:60), producing 200 mg (33%) of **13** as white crystals: (Rf = 0.15, hexane/AcOEt 40:60); IR (cm^−1^): 3284, 2930, 1737, 1655, 1454, 1369, 1226, 1177, 1022, 746; ^1^H-NMR (600 MHz; CDCl_3_): δ 7.60 (1H, d, *J* = 7.6 Hz, CH-4), 7.43 (1H, d, *J* = 8.2 Hz, CH-7), 7.31–7.23 (2H, m, CH-6, CH-5), 6.34 (1H, bs, NH), 5.28 (2H, s, CH_2_-12), 4.67 (2H, d, *J* = 5.7 Hz, CH_2_-8), 2.04 (3H, s, CH_3_-15), 2.01 (3H, s, CH_3_-11); ^13^C-NMR (150 MHz; CDCl_3_): δ 171.4 (C-10), 169.9 (C-14), 154.1 (C-7a), 152.6 (C-2), 127.7 (C-3a), 124.8 (CH-6), 123.1 (CH-5), 119.7 (CH-4), 112.4 (C-3), 111.3 (CH-7), 56.4 (CH_2_-12), 35.0 (CH_2_-8), 23.1 (CH_3_-11); 21.0 (CH_3_-15).

*N-((3-formylbenzofuran-2-yl)methyl)acetamide* (**14**) [27,28]. To a solution of **13** (200 mg, 0.766 mmol) in 8 mL of MeOH, 530 mg (3.83 mmol) of K_2_CO_3_ was added and stirred at rt for 30 min. Then, 10 mL of water was added to dissolve the excess carbonate before extraction with DCM (3 × 20 mL). The organic phase was treated with Na_2_SO_4_ and filtered, and the solvent was evaporated to generate *N*-((3-(hydroxymethyl)benzofuran-2-yl) methyl) acetamide (160 mg, 95%), which was used in the ensuing oxidation reaction. ^1^H-NMR (600 MHz; CDCl_3_): δ 7.60 (1H, d, *J* = 7.1 Hz, CH-4), 7.40 (1H, d, *J* = 7.1 Hz, CH-7), 7.31–7.23 (2H, m, CH-6, CH-5), 6.23 (1H, bs, NH), 4.87 (2H, s, CH_2_-12), 4.57 (2H, d, *J* = 6.1 Hz, CH_2_-8), 3.94 (1H, bs, OH) and 1.98 (3H, s, CH_3_-11). In 20 mL of DCM, 150 mg (0.685 mmol) of the above product was dissolved, followed by the addition of 295 mg (1.37 mmol) of pyridinium chlorochromate (PCC) and 147 mg of AcONa (1.8 mmol). The mixture was stirred at rt under nitrogen atmosphere and the progress of the reaction was monitored by TLC. Subsequently, the crude reaction mixture was filtered through neutral Al_2_O_3_ by rinsing with 30 mL acetone, and the filtrate was concentrated and subjected to column chromatography on silica gel (hexane/AcOEt, 50:50 to 30:70), resulting in 70 mg (47%) of **14** as a yellow solid: (Rf = 0.24, hexane/AcOEt 40:60); m.p. 153–155 °C; ^1^H-NMR (600 MHz, CDCl_3_): δ 10.38 (1H, s, CHO-13), 8.12 (1H, d, *J* = 6.5 Hz, CH-4), 7.49 (1H, d, *J* = 6.6 Hz, CH-7), 7.40–7.35 (2H, m, CH-6, CH-5), 6.26 (1H, bs, NH), 4.87 (2H, d, *J* = 6 Hz, CH_2_-8), 2.05 (3H, s, CH_3_-11); ^13^C-NMR (150 MHz, CDCl_3_): δ 185.3 (C-12), 169.9 (C-10), 162.9 (C-2), 154.1 (C-7a), 126.0 (CH-6), 124.9 (CH-5), 124.5 (C-3a), 121.8 (CH-4), 118.2 (C-3), 111.3 (CH-7), 35.4 (CH_2_-8); 23.1 (CH_3_-11).

*Methyl 2-(acetamidomethyl)benzofuran-3-carboxylate* (**2**) [29,30,31]. To 70 mg aldehyde **14** (0.32 mmol) in 5 mL MeOH, we added 105 mg KCN (1.6 mmol) and 0.036 mL glacial acetic acid (0.64 mmol), and the mixture was stirred at rt for 1 h. After adding 78 mg NaCN (1.6 mmol) and 36 µL glacial acetic acid (0.64 mmol) and stirring continuously for 30 min, 560 mg MnO_2_ (6.4 mmol) was added and the mixture was stirred for another 2 h at rt before being filtered through celite and concentrated under vacuum. The residue was diluted with water and extracted with DCM (3 × 15 mL), and the organic phase was washed with water (1 × 30 mL) and brine (1 x 30 mL), then dried with anhydrous Na_2_SO_4_ and concentrated under vacuum. The crude mixture was subjected to column chromatography on silica gel (hexane/AcOEt, 35:65) to produce methyl ester **2** as beige crystals: (Rf = 0.4, hexane/AcOEt 30:70); m.p. 128–130 °C; IR (cm^−1^): 3290, 3070, 1714, 1655, 1595, 1544, 1452, 1238, 1175, 1107, 1073, 752; ^1^H-NMR (600 MHz; CDCl_3_): δ 7.94 (1H, m, CH-4), 7.48 (1H, m, CH-7), 7.34–7.30 (2H, m, CH-6, CH-5), 6.44 (1H, bs, NH), 4.91 (2H, d, *J* = 6.1 Hz, CH_2_-8), 3.97 (3H, s, CH_3_-16), 2.02 (3H, s, CH_3_-12); ^13^C-NMR (150 MHz, CDCl_3_): δ 169.8 (C-10), 164.8 (C-13), 161.7 (C-2), 153.7 (C-7a), 125.3 (C-3a), 125.3 (CH-6), 124.2 (CH-5), 122.0 (CH-4), 111.5 (CH-7), 110.2 (C-3), 51.8 (CH_3_-16), 36.4 (CH_2_-8), 23.2 (CH_3_-12); HRMS (ESI^+^): calcd for C_13_H_13_NO_4_Na [M + Na]^+^, 270.0692; found, 270.0692.

*Methyl 2-(acetamidomethyl)-2,3-dihydrobenzofuran-3-carboxylate* (***rac*-2a**). Yellow Oil: (Rf = 0.09 Hex/AcOEt 1:1); ^1^H-NMR (600 MHz, CDCl_3_): δ 7.36 (1H, d, *J* = 7.5 Hz, CH-4), 7.20 (1H, t, *J* = 7.5 Hz, CH-6), 6.91 (1H, t, *J* = 7.5 Hz, CH-5), 6.80 (1H, d, *J* = 7.8 Hz, CH-7); 5.91 (1H, bs, NH), 5.17 (1H, td, *J* = 6.5, 4.1 Hz, CH-2), 4.09 (1H, d, *J* = 7.2 Hz, CH-3), 3.79 (3H, s, CH_3_-16), 3.72 (1H, m, CH_2_-8A), 3.59 (1H, m, CH_2_-8B), 1.98 (3H, s, CH_3_-12); ^13^C-NMR (150 MHz; CDCl_3_): δ 170.9 (C-13), 170.5 (C-10), 153.7 (C-7a), 129.6 (CH-6), 125.6 (CH-4), 123.9 (C-3a), 121.1 (CH-5), 109.8 (CH-7), 83.2 (CH-2), 52.7 (CH_3_-16), 49.9 (CH-3), 42.5 (CH_2_-8), 23.2 (CH_3_-12); The racemic mixture was examined by HPLC on a Chiralcel OJ-H column (4.6 × 250 mm; Chiral technologies Europe, bd girthier d’andernach, Illkirch, France), with the mobile phase of an 80:20 Hex:i-PrOH system at a flow rate of 0.6 mL/min and at 25 °C, and with the UV light detector at λ = 279 nm. *R*t_1_ = 13.31, *R*t_2_ = 16.86 min; HRMS (ESI^+^): calculated for C_13_H_15_NO_4_, 249.10; found 250.1082 (M+1). 

### 3.3. Theoretical Calculations 

Structures **1** and **2** were optimized using the B3LYP level of theory with the 6-31G basis on Gaussian 09 software (Gaussian Inc., Quinnipiac St Bldg 40 Wallingford, CT 06492 USA). Single-point energies for the ionic structures (anion and cation) were calculated at the same level of theory with UB3LYP/6-31G [37].

#### Indices of Global and Local Reactivity

All above calculations were performed on Gaussian 09 software (Gaussian Inc., Quinnipiac St Bldg 40 Wallingford, CT 06492 USA), which allows for the calculation of the first ionization potential (***I***) and the electron affinity (***A***) to obtain the global parameters. The chemical potential (**µ**) was calculated as **µ** = –(***I*** + ***A***)/2, global hardness (**η**) as **η** = (*I* − ***A***), global softness (**S**) as **S** = 1/**η**, the electrophilicity index (**ω**) as **ω** = **µ**^2^/2 **η**, the electron donating power (**ω^−^**) as **ω^−^** = (3***I*** + ***A***)^2^/16 (***I*** − ***A***), the electron accepting power (**ω^+^**) as **ω^+^** = (***I*** + *3**A***)^2^/16 (***I*** − ***A***), and the nucleophilicity index (**N**) as **N**_(Nu)_ = *E*_HOMO (Nu)_ (eV) − *E*_HOMO (TCE)_ (eV). The condensed FFs were evaluated by the following equations: (***f_k_*^+^**) = [q*_k_* (N + 1) − q*_k_* (N)] for reaction with nucleophiles, and (***f_k_*^−^**) = [q*_k_* (N) − q*_k_* (N − 1)] for reaction with electrophiles. Another local descriptor is local softness, which is the product of the condensed FF (***f_k_*^+/−^)** multiplied by the global softness (**S**).

For each atom in the ***k*** position of a molecule, **s*_k_*^+^** = ***f_k_^+^*** **S** expresses the susceptibility to receiving a local nucleophilic attack, whereas **s*_k_^−^*** = ***f_k_^−^ S*** indicates the tendency to make a nucleophilic attack. The local electrophilicity (**ω*_k_***) is calculated as **ω*_k_*** = **ω*f_k_*^+^** and the local nucleophilicity (**N*_k_***) as **N*f_k_***. The electron population for calculating the FFs was based on the formulation of the quantum theory of atoms in molecules (QTAIM). The wave function was calculated for each of the neutral and ionic systems, using the geometry optimized for the neutral molecule. By using the wave function value, the electron population was determined on AIM 2000 software (Version 2.37, Chemical advice by R.F.W. Bader, McMaster University, Hamilton, Canada). The electrophilic (***P_k_^+^***) and nucleophilic (***P_k_*^−^**) Parr functions were defined through the analysis of the Mulliken atomic spin density (ASD) of the radical anion and the radical cation by single-point energy calculations over the optimized neutral geometries, employing the unrestricted UB3LYP formalism for radical species. 

## 4. Conclusions

Compounds ethyl 3-(acetamidomethyl)benzofuran-2-carboxylate and methyl 2-(acetamidomethyl)benzofuran-3-carboxylate (**1** and **2,** respectively) were synthesized with overall yields of 23 and 5.4%, respectively. They were characterised by their FT-IR, NMR, and HRMS spectral data. Since obtaining the 2,3-dihydroderivatives was more difficult for **1** than **2**, the catalytic reduction conditions were optimized with Pd/C at 10% for the former. The reduction of **1** produced the hydrogenated derivatives ***rac*-1a** and **1b** as the main products. When the concentration of the catalyst increased, the reaction produced the octahydroderivative **1c**. Conversely, the hydrogenation of **2** only led to ***rac*-2a** as the main product. ^1^H-NMR was crucial for evaluating the relative proportions of each product in the reduction experiments. These results were corroborated by theoretical calculations of global and local reactivity, which indicated the electrophilic behavior of **1**. Whereas three olefinic carbons in **1** are susceptible to reduction (C3, C4, and C6), this susceptibility only exists with one olefinic carbon (C2) in **2**. The theoretical calculations, NMR spectra, and the resonance structures clearly revealed the most susceptible moieties for a nucleophilic attack, thus explaining the selectivity in reduction. 

## Figures and Tables

**Table 1:** Chemical shifts (δ, ppm) in the proton nuclear magnetic resonance (^1^H-NMR) spectra of the methyls in compound **1** and its hydrogenated products (**1a**–**1c**), and in compound **2** and its hydrogenated derivative **2a**.

Compound	Structure	δ NH-14		δ CH_3_-17		δ CH_3_-12	
**1**	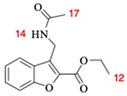	6.36 ^a^	-	1.96 ^a^	1.94 ^b^	1.45 ^a^	1.41 ^b^
**1a**	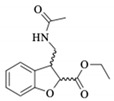	5.73 ^a^	-	1.87 ^a^	1.92 ^b^	1.34 ^a^	1.31 ^b^
**1b**	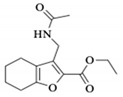	6.61 ^a^	-	1.94 ^a^	1.92 ^b^	1.38 ^a^	1.33 ^b^
**1c**	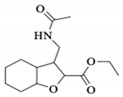	6.27 ^a^	-	1.96 ^a^	1.92 ^b^	1.34 ^a^	1.31 ^b^
		**δ NH-9**		**δ CH_3_-16**		**δ CH_3_-12**	
**2**	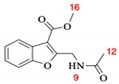	6.45 ^a^	-	3.98 ^a^	-	2.02 ^a^	-
**2a**	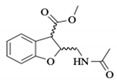	5.93 ^a^	-	3.78 ^a^	-	1.98 ^a^	-

Chemical shifts refer to internal Tetramethylsilane TMS at 600 MHz. (^a^ CDCl_3_, ^b^ CD_3_OD.).

**Table 2:** Reaction conditions for the catalytic hydrogenation of compounds **1** and **2** with 10% Pd/C, at room temperature (rt) and 65 psi of H_2_ pressure.

Entry ^a^	Substrate (S) (mg/mmol)	Weight Ratio (S/Catal)	Concentration [mmol]	Time (h)	Yield (%)				
					1	*rac*-1a	1b	1c	
1	50/0.19	1/0.5	76	24	55	34	7	4	^c^
2	50/0.19	1/1	76	24	23	54	10	13	^c^
3	50/0.19	1/1.5	76	24	13	42	14	32	^c^
4	50/0.19	1/2	38	24	12	21	22	46	^c^
5	50/0.19	1/0.5	38	44	77	19	4	---	^c^
6	15/0.06	1/1	38	44	30	55	9	6	^c^
7	100/0.38	1/2	14	46	35	26	25	---	^c^
8	100/0.38	1/2.4	82	48	---	13	24	63	^c, d^
9	71/0.27	1/1	16	96	88	7	5	---	^c^
10	100/0.38	1/4	14	24	7	47	31	15	^c, d^
					**2**	***rac*-2a**			
11	25/0.10	1/0.3	45	44	100	---	---	---	^c^
12	15/0.06	1/1	75	24	20	80	---	---	^c^
13 ^b^	26/0.10	1/1	75	24	12	88	---	---	^c^

All reactions were performed with ^a^ EtOH or ^b^ MeOH as solvent. ^c^ Ratios were determined by ^1^H-NMR of the crude mixture. ^d^ Yield of products isolated by chromatography.

**Table 3:** Global indices of reactivity: ionization potential (***I***), electron affinity (***A***), chemical potential (***µ***), global hardness (**η**), global softness (***S***), electrophilicity index (**ω**), nucleophilicity index (**N**), electron donating power (**ω^−^**), and electron accepting power (**ω^+^**).

Comp.	*I*(eV)	*A*(eV)	*µ*(eV)	η(eV)	*S*(1/eV)	ω(eV)	N (eV)	ω^−^(eV)	ω^+^(eV)
**1**	8.031	0.136	−4.083	7.895	0.1267	1.056	1.087	4.647	0.564
**2**	7.993	0.067	−4.030	7.926	0.1261	1.025	1.125	4.560	0.530

The Highest Occupied Molecular Orbital (HOMO) energy of tetracyanoethylene (TCE) is −0.3351 au (27.21) = 9.118 eV at the same level of theory.

**Table 4:** The electrophilic (***f_k_*^+^**) and nucleophilic (***f_k_*^−^**) Fukui functions, as well as the electrophilic and nucleophilic local softness **s*_k_*^+^** and **s*_k_*^−^,** and the corresponding local electrophilicity index **ω*_k_*^a^** at different positions in the compound. Also presented are the electrophilic (***P_k_*^+^**) and nucleophilic (***P_k_*^−^**) Parr functions, and the corresponding local electrophilicity index redefined as **ω*_k_*^b^**.

Comp. *k*		*f_k_* ^+^	*f_k_* ^−^	s*_k_*^+^	s*_k_*^−^	ω*_k_* ^a^ eV	*P_k_^+^* ρ_s_^ra^(k)	*P_k_^−^* ρ_s_^rc^(k)	ω*_k_* ^b^ eV
**1**	C2	0.0350	0.05505	0.0044	0.0070	0.03696	0.1170	0.2227	0.1236
	C3	0.0646	0.02911	0.0082	0.0037	0.06822	0.2372	0.1198	0.2505
	C3a	0.0059	0.01383	0.0007	0.0018	0.00623	−0.0763	−0.0261	−0.0806
	C4	0.0451	0.04218	0.0057	0.0053	0.04763	0.173	0.2393	0.1827
	C5	0.0255	0.03098	0.0032	0.0039	0.02693	−0.0768	−0.0856	−0.0811
	C6	0.0462	0.04162	0.0059	0.0053	0.04879	0.1407	0.1436	0.1486
	C7	0.0373	0.04826	0.0047	0.0061	0.03939	0.0237	0.1308	0.025
	C7a	0.0068	0.00060	0.0009	0.0001	0.00718	0.0199	−0.0388	0.021
**2**	C2	0.0935	0.03574	0.0115	0.0044	0.09584	0.5483	0.1473	0.562
	C3	0.0270	0.03411	0.0033	0.0042	0.02768	−0.0608	0.1408	−0.0623
	C3a	0.0033	0.01697	0.0004	0.0021	0.00338	−0.0145	0.0321	−0.0149
	C4	0.0327	0.03945	0.0040	0.0049	0.03352	0.0749	0.1899	0.0768
	C5	0.0302	0.02737	0.0037	0.0034	0.03096	−0.0045	−0.1063	−0.0046
	C6	0.0366	0.04421	0.0045	0.0054	0.03752	0.0140	0.2217	0.0144
	C7	0.0405	0.04005	0.0050	0.0049	0.04151	0.0779	0.0442	0.0798
	C7a	−0.0107	0.01281	−0.0013	0.0016	−0.0109	−0.0441	0.0156	−0.0452

The electron population for calculating the Fukui functions was based on the theory of atoms in molecules (AIM). The Parr functions were obtained through the analysis of the Mulliken atomic spin density (ASD) of the radical anion and the radical cation. ^a^ **ω*_k_*** was calculated with the Fukui functions; ^b^ **ω*_k_*** was calculated with the Parr functions.

## Supplementary Materials

The following are available online at https://www.mdpi.com/1420-3049/24/11/2061/s1, Figures S1–S22 describe NMR data for all compounds. Figures S23–S28 provide HRMS spectra for **1**, ***rac*-1a**, **1b**, **1c**, **2**, and ***rac*-2a**. Figures S29–S33 describe HPLC data for **1**, ***rac*-1a**, **1b**, **2** and ***rac*-2a**. Figures S34 and S35 depict optimized geometries of **1** and **2**. Tables S1–S8 describe computational data.
