# Supplementary material for: Quantum Reality in the Selective Reduction of a Benzofuran System
Source: Molecules. 2019 May 30;24(11):2061. doi: 10.3390/molecules24112061 (PMC6600454; doi:10.3390/molecules24112061)
Supplement: Supplementary file 1 [file molecules-24-02061-s001.pdf]

Supplementary Material

Quantum reality in the selective reduction of a benzofuran system.

Arturo Coaviche-Yoval [1], Erik Andrade-Jorge [2,3], Cuauhtémoc Pérez-González [4], Héctor Luna[4], Ricardo Tovar-Miranda [5,*], José G. Trujillo-Ferrara[2,*]

[1] Doctorado en Ciencias Biológicas y de la Salud, Universidad Autónoma Metropolitana-Unidad Xochimilco, 04960 Cd. México, Mexico; a.cy2@hotmail.com (A.C.-Y.)
[2] Departamento de Bioquímica, Sección de Estudios de Posgrado e Investigación, Escuela Superior de Medicina, Instituto Politécnico Nacional, 11340 Cd. México, Mexico; andrade136@hotmail.com (E.A.-J.); jtrujillo@ipn.mx (J.G.T.-F.)
[3] Unidad de Investigación en Biomedicina, Facultad de Estudios Superiores-Iztacala, Universidad Nacional Autónoma de México. Av. de los Barrios 1, Los Reyes Iztacala, Tlalnepantla, 54090, Estado de México, Mexico; andrade136@hotmail.com (E.A.-J.)
[4] Departamento de Sistemas Biológicos, Universidad Autónoma Metropolitana-Unidad Xochimilco, 04960 Cd. México, Mexico; cperezg@correo.xoc.uam.mx (C.P.-G.); lchm1964@correo.xoc.uam.mx (H.L.)
[5] Instituto de Ciencias Básicas, Universidad Veracruzana, 91190 Xalapa, Veracruz, Mexico; rtovar@uv.mx (R.T.-M.)
* Correspondence: jtrujillo@ipn.mx; Tel.: +52-55-5729-6000 (ext. 62747) (J.G.T.-F.); rtovar@uv.mx; Tel.: +52-22-8841-8900 (ext. 13930) (R.T.-M.)

Table of Contents

NMR spectra 2

HRMS spectra for 1, *rac*-1a, 1b, 1c, 2 and *rac*-2a 13

HPLC Data for 1, *rac*-1a, 1b, 2 and *rac*-2a 15

Coordinates and Energies 16

Energies and atomic charges in the frame B3LYP/6-31+G* of the molecule 1 22

Energies and atomic charges in the frame B3LYP/6-31+G* of the molecule 2 23

NMR spectra

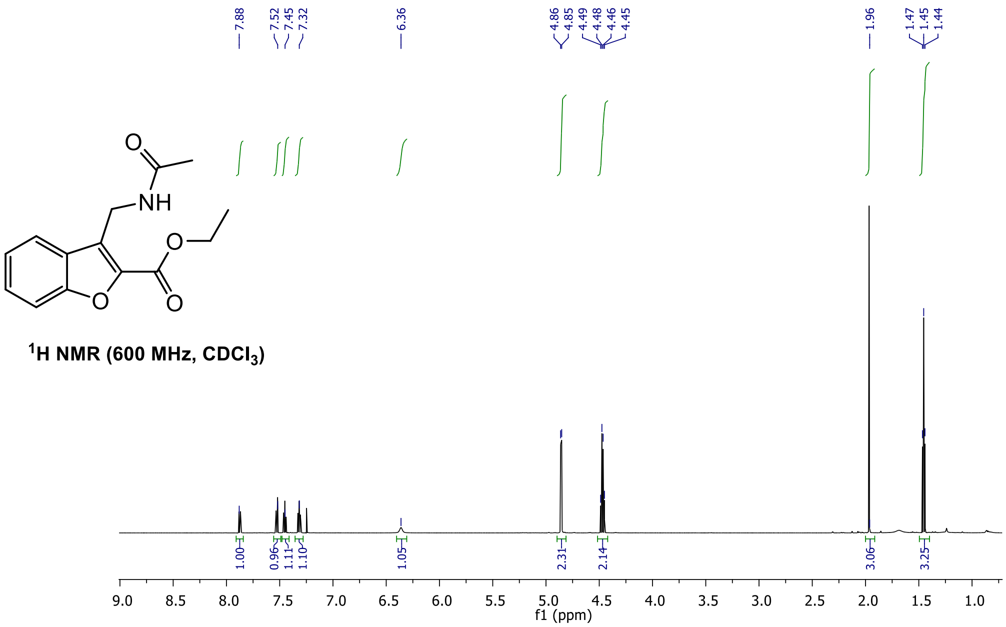

Figure S1: ^{1}H NMR spectrum of **1**

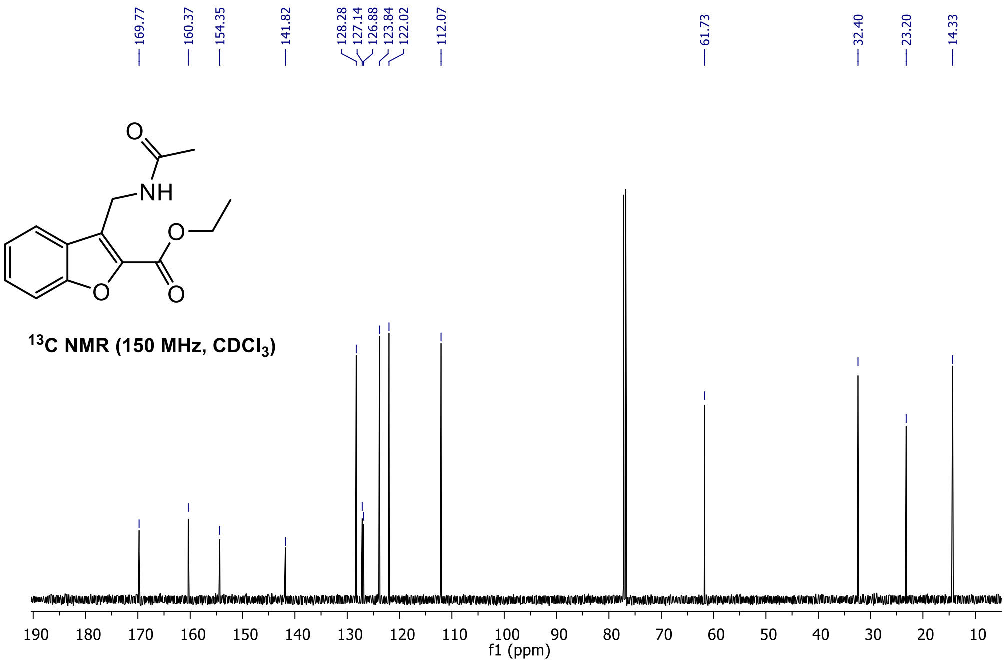

Figure S2: ^{13}C NMR spectrum of **1**

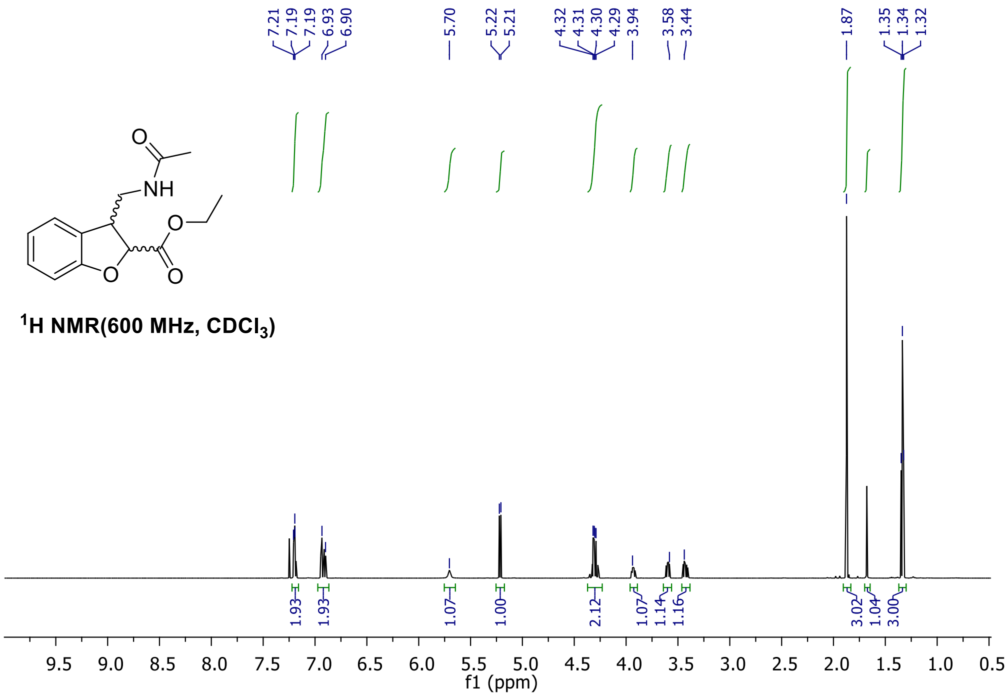

Figure S3: ^{1}H NMR spectrum of ***rac*-1a**

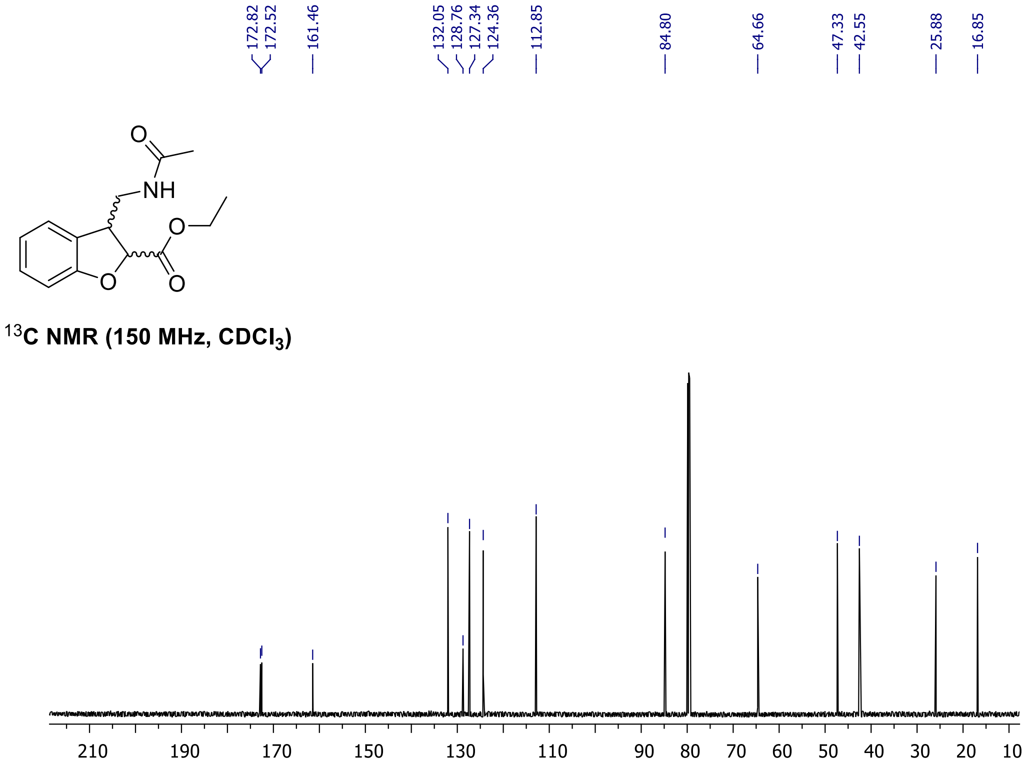

Figure S4: ^{13}C NMR spectrum of ***rac*-1a**

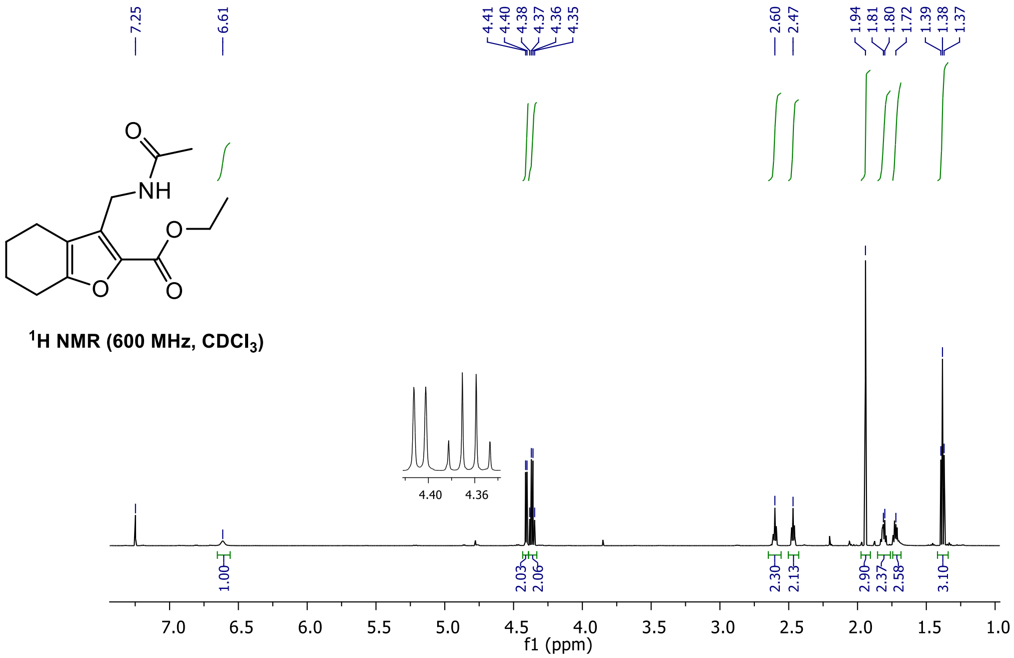

Figure S5: ^{1}H NMR spectrum of **1b**

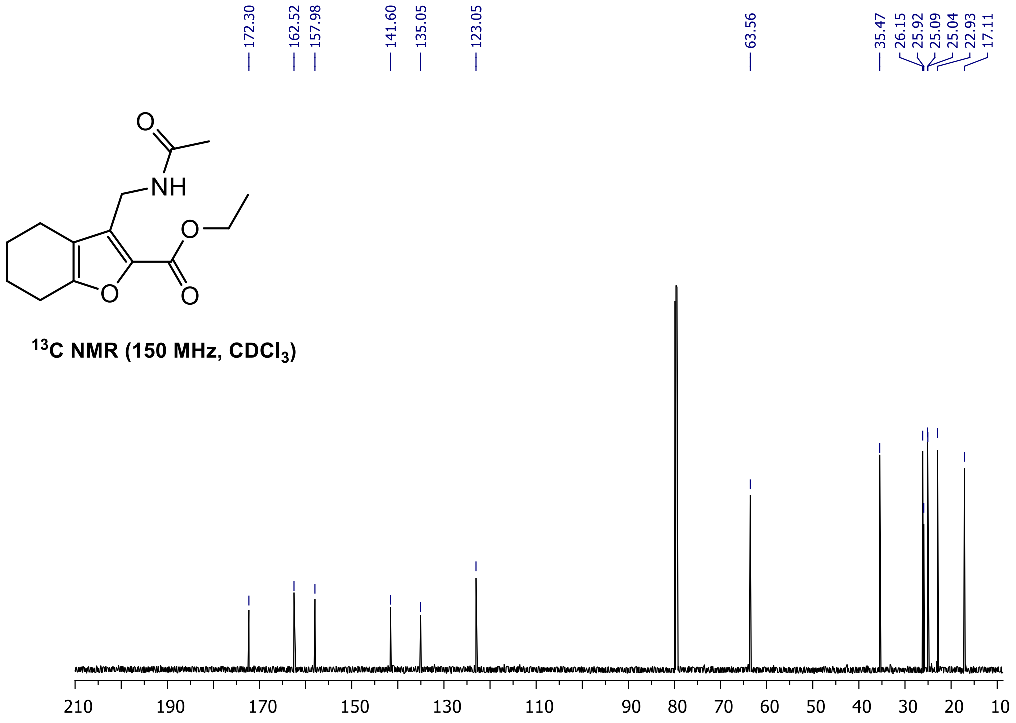

Figure S6: ^{13}C NMR spectrum of **1b**

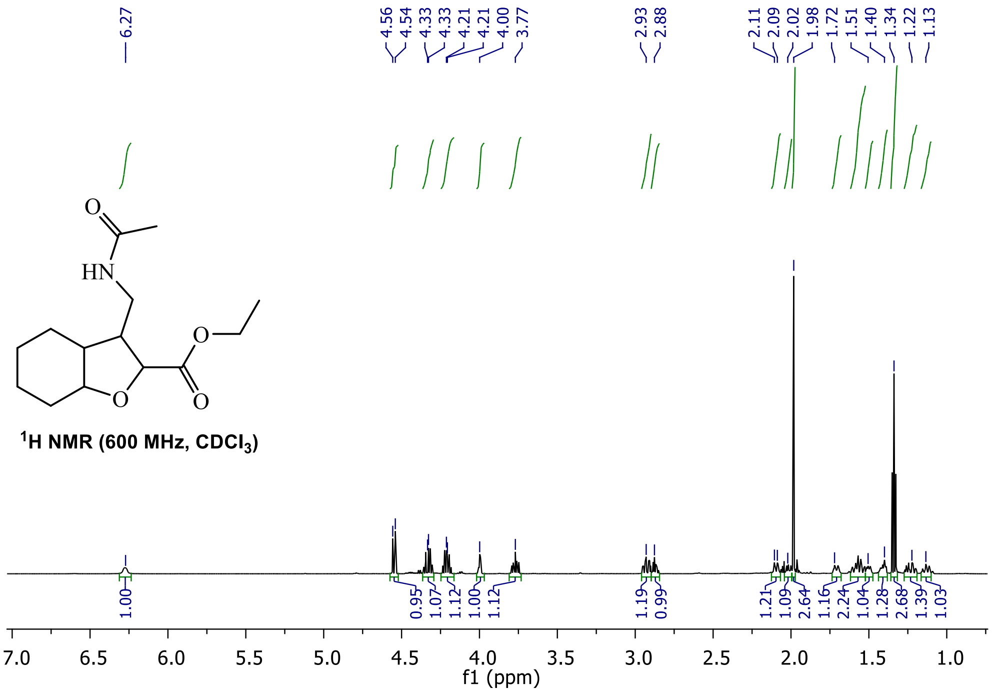

Figure S7: ^{1}H NMR spectrum of **1c**

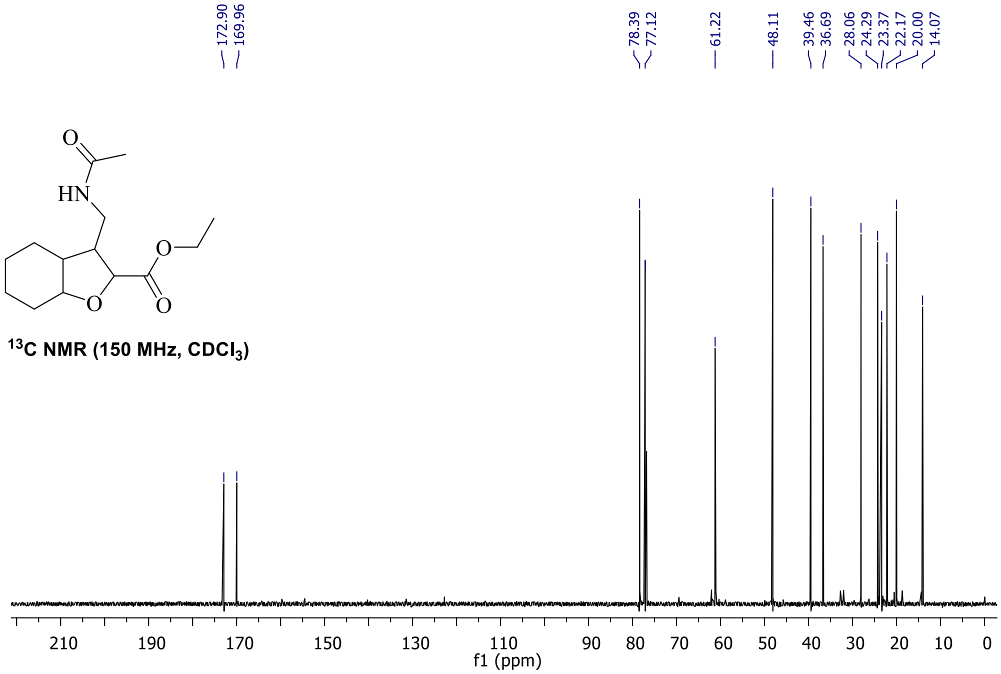

Figure S8: ^{13}C NMR spectrum of **1c**

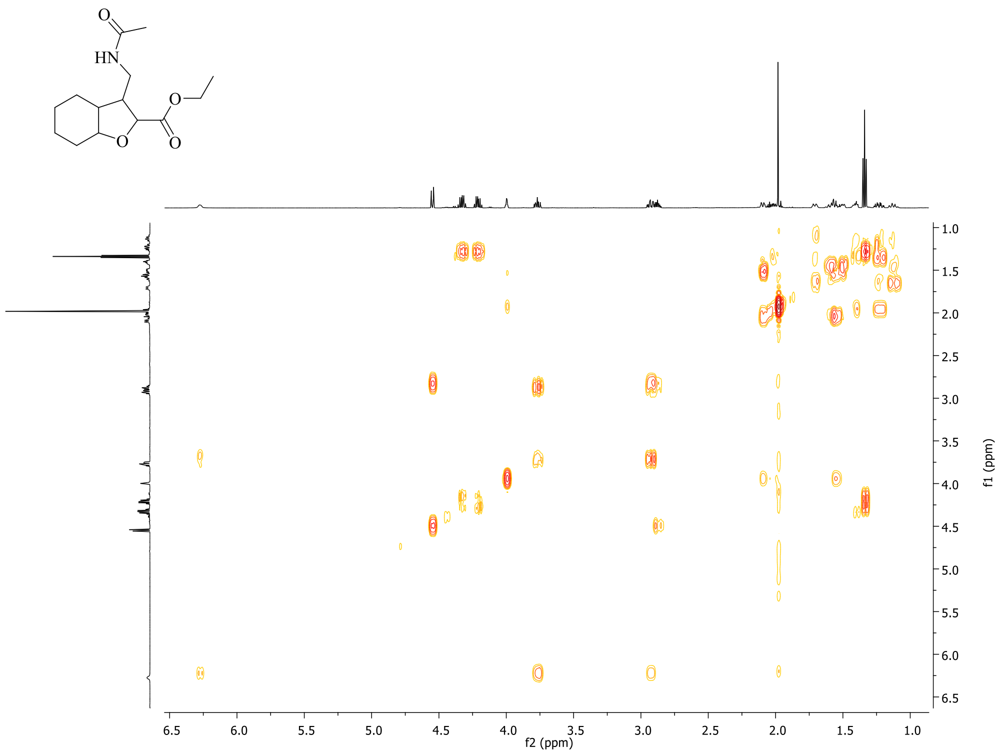

Figure S9: gCOSY spectrum of **1c**

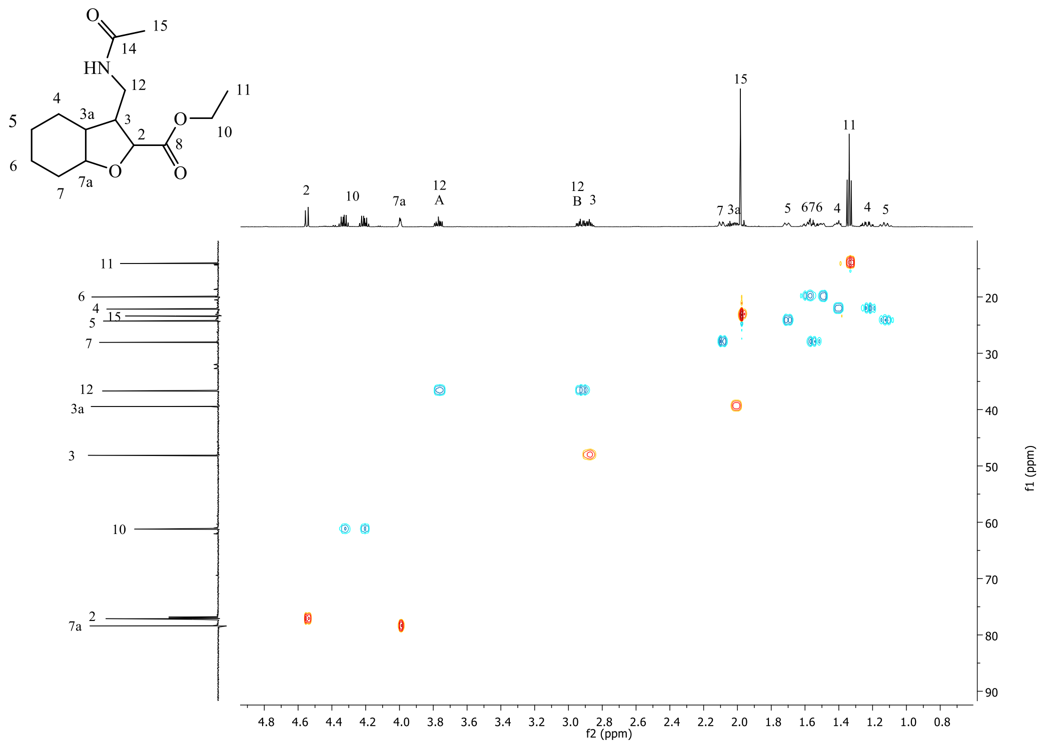

Figure S10: gHSQC spectrum of **1c**

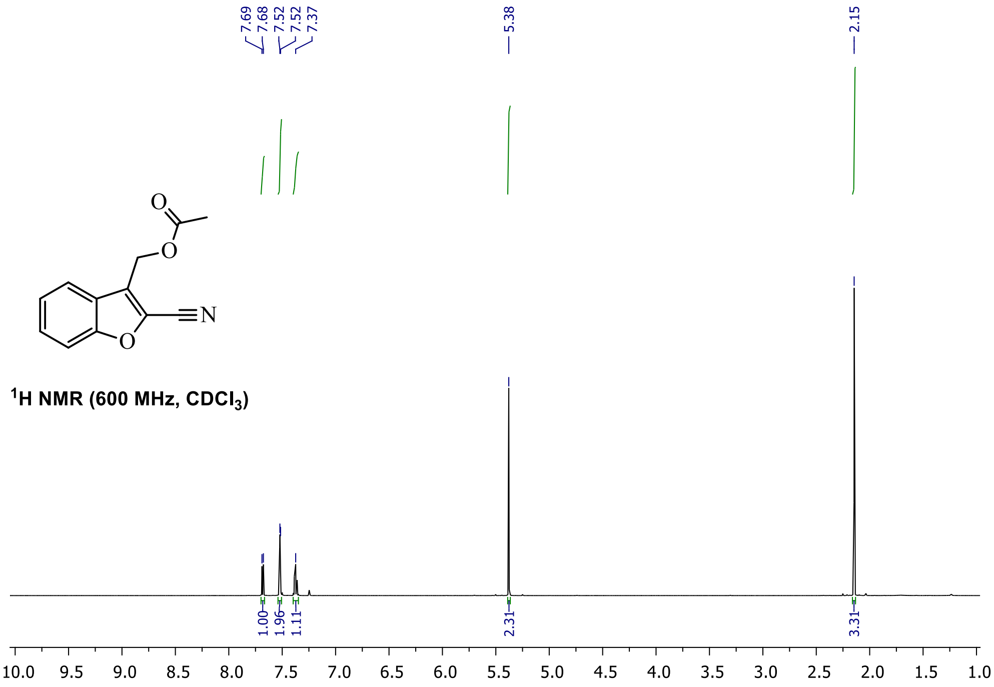

Figure S11: ^{1}H NMR spectrum of **11**

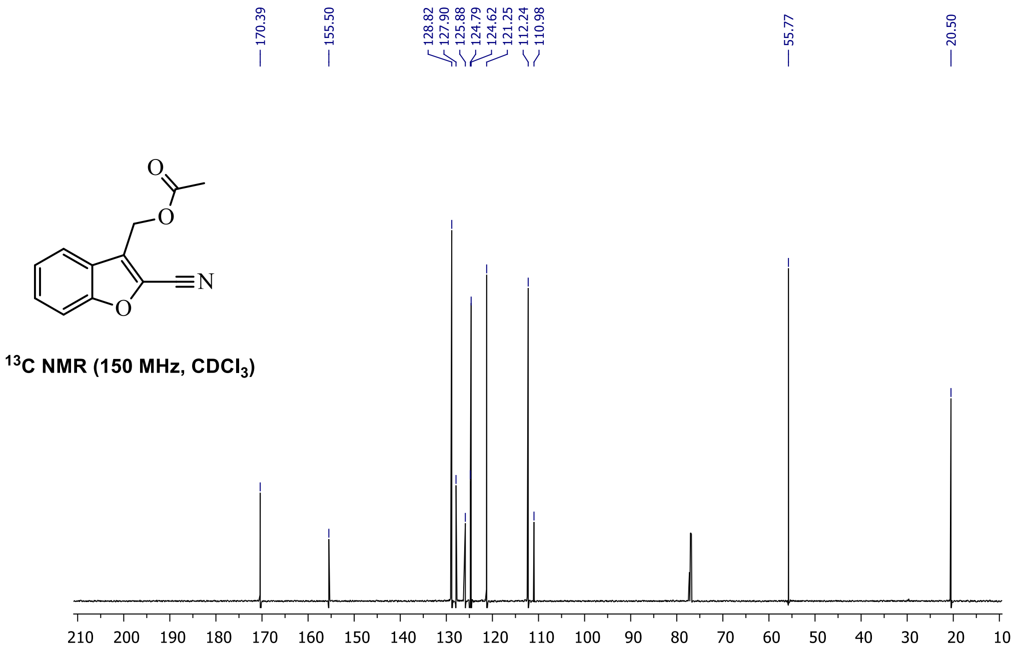

Figure S12: ^{13}C NMR spectrum of **11**

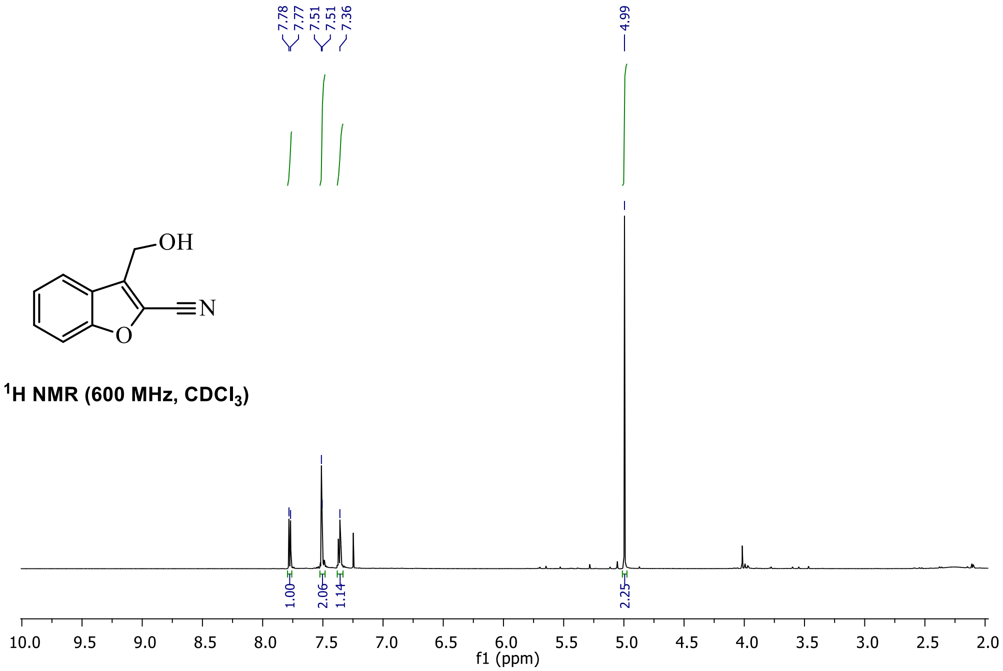

Figure S13: ^{1}H NMR spectrum of **12**

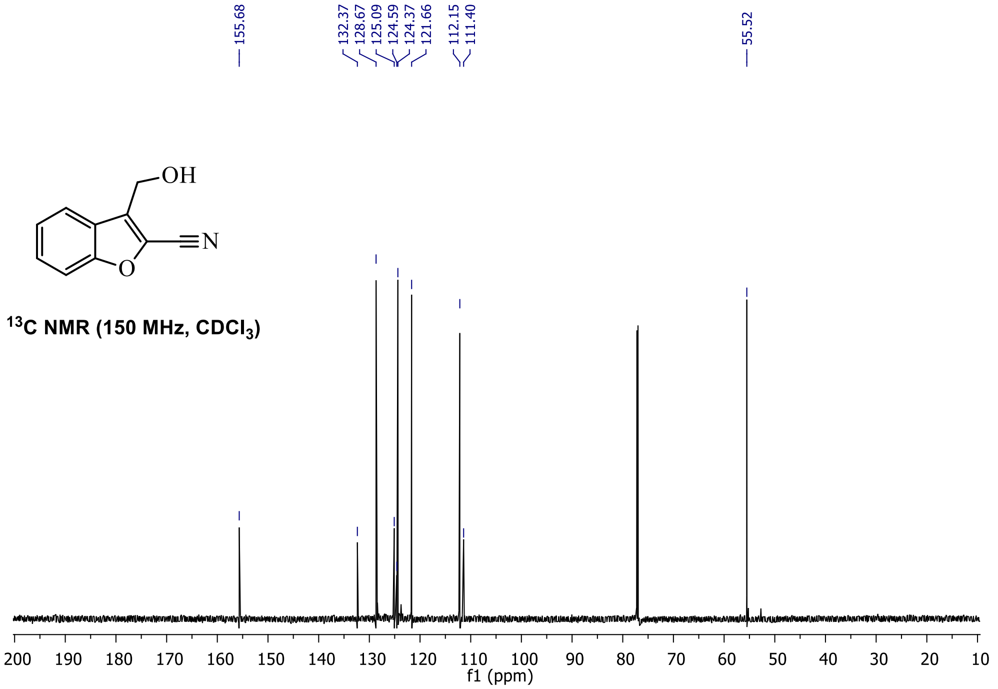

Figure S14: ^{13}C NMR spectrum of **12**

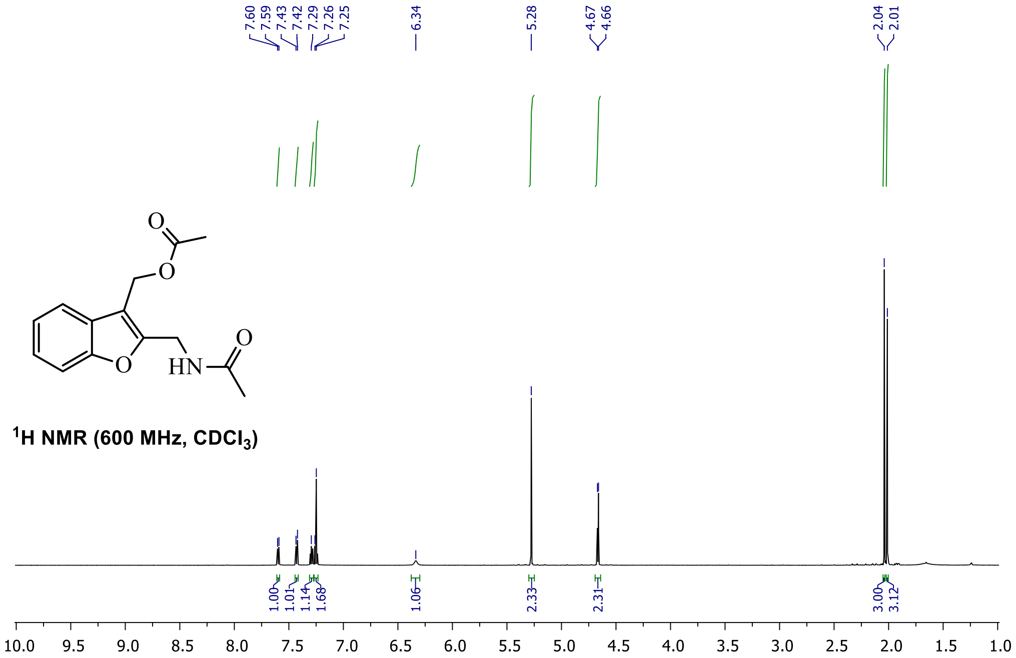

Figure S15: ^{1}H NMR spectrum of **13**

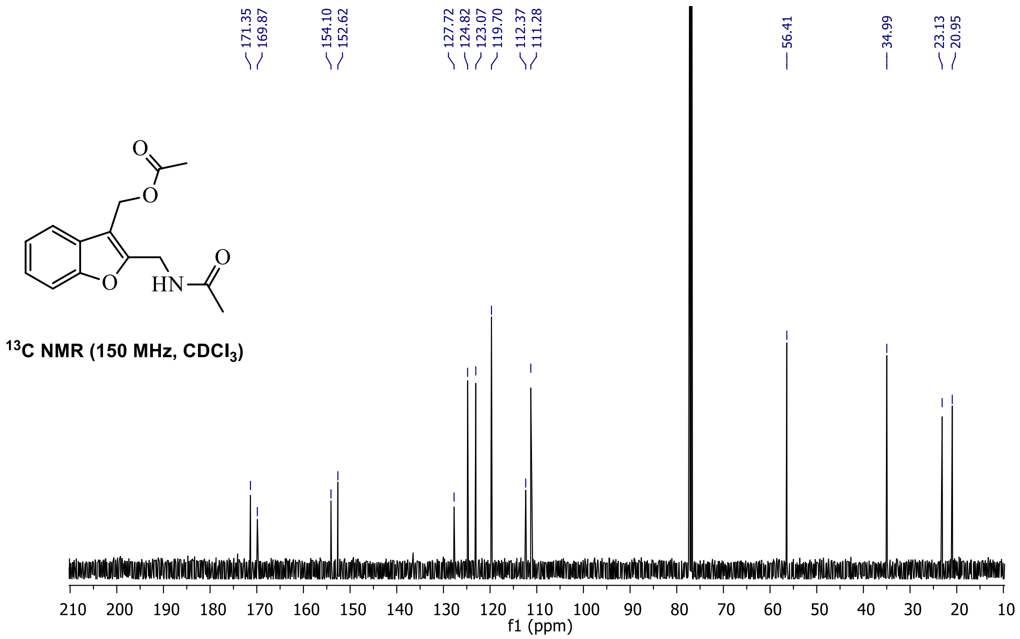

Figure S16: ^{13}C NMR spectrum of **13**

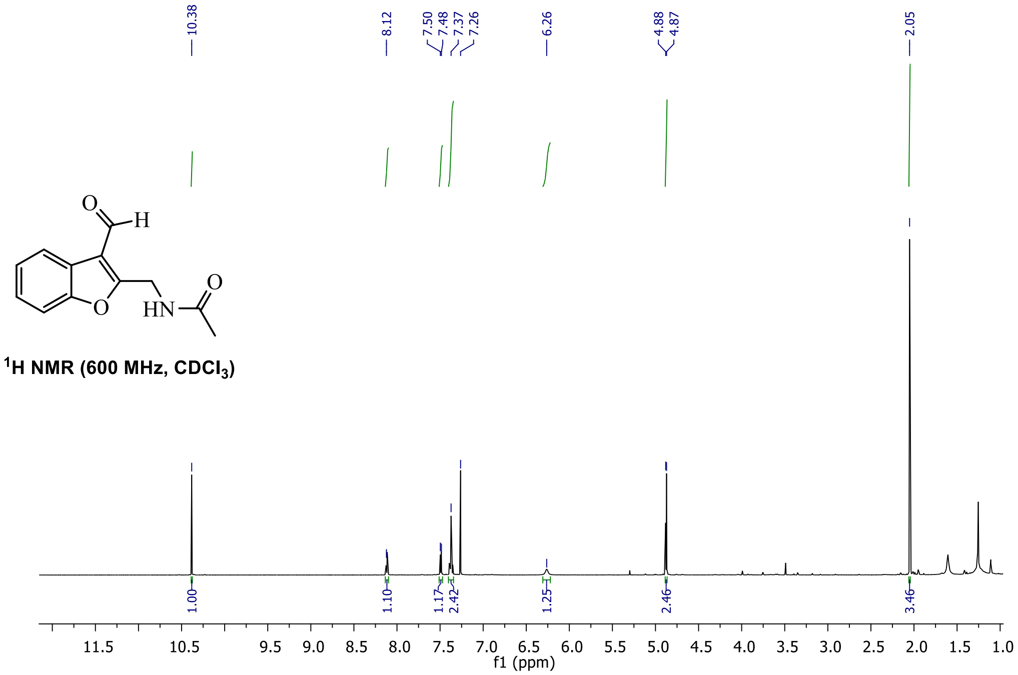

Figure S17: ^{1}H NMR spectrum of **14**

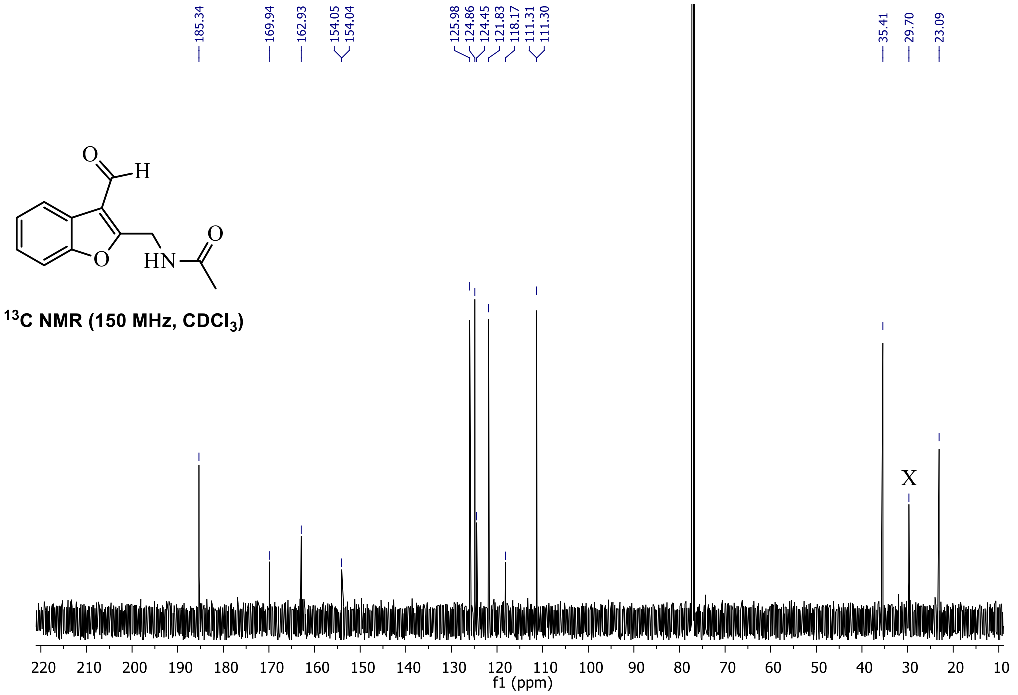

Figure S18: ^{13}C NMR spectrum of **14**

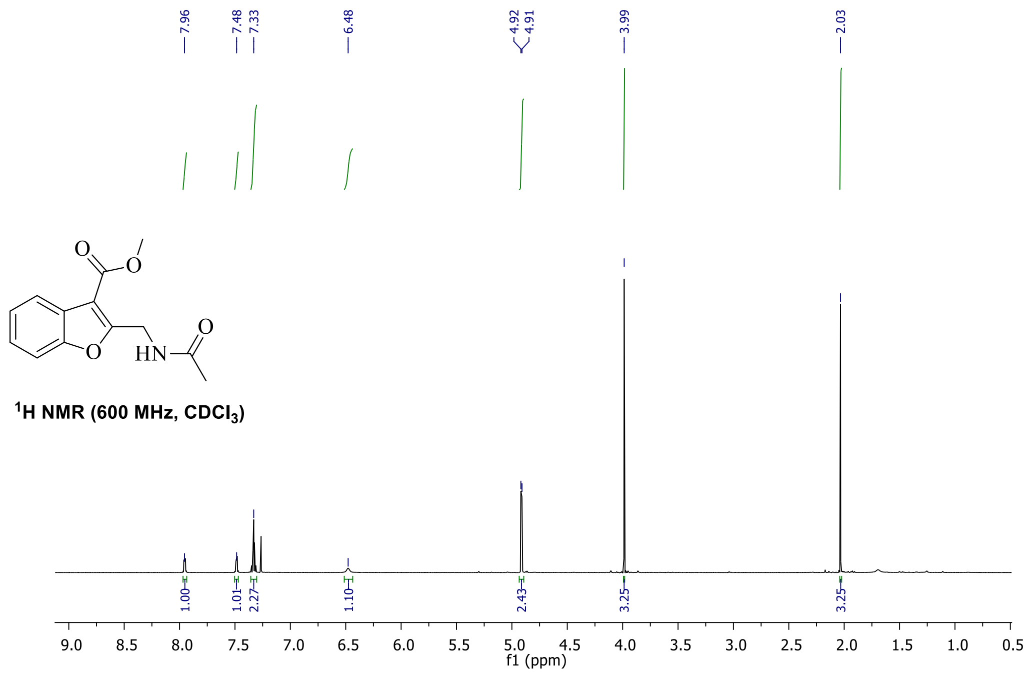

Figure S19: ^{1}H NMR spectrum of **2**

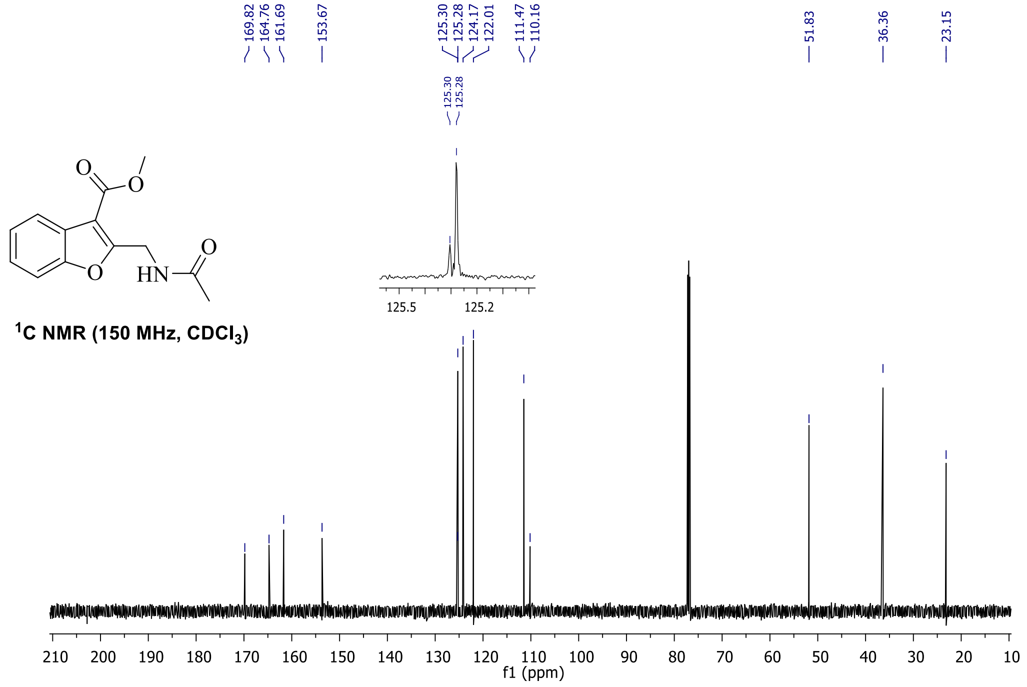

Figure S20: ^{13}C NMR spectrum of **2**

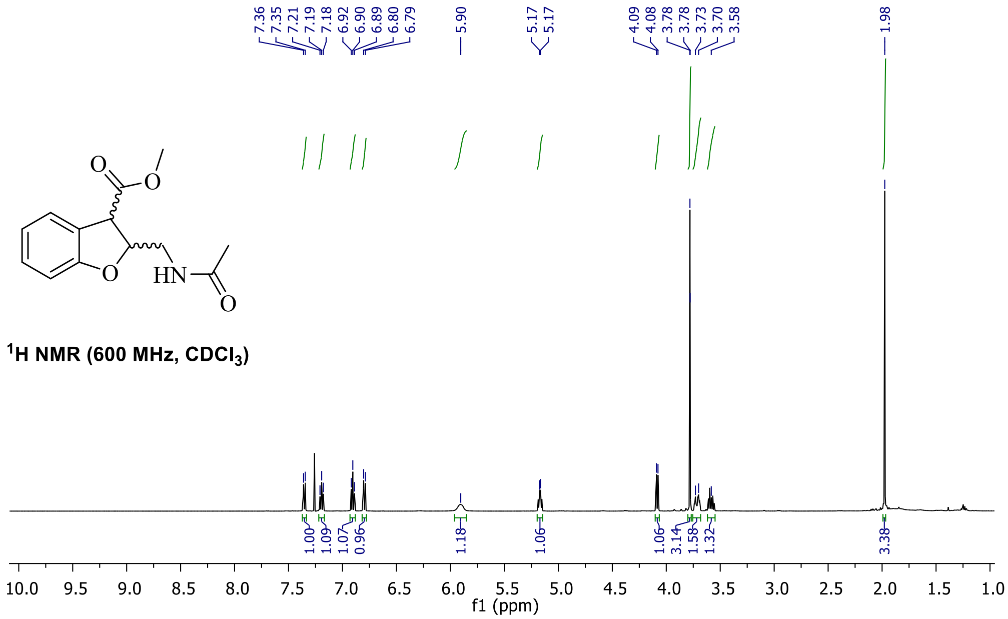

Figure S21: ^{1}H NMR spectrum of ***rac*-2a**

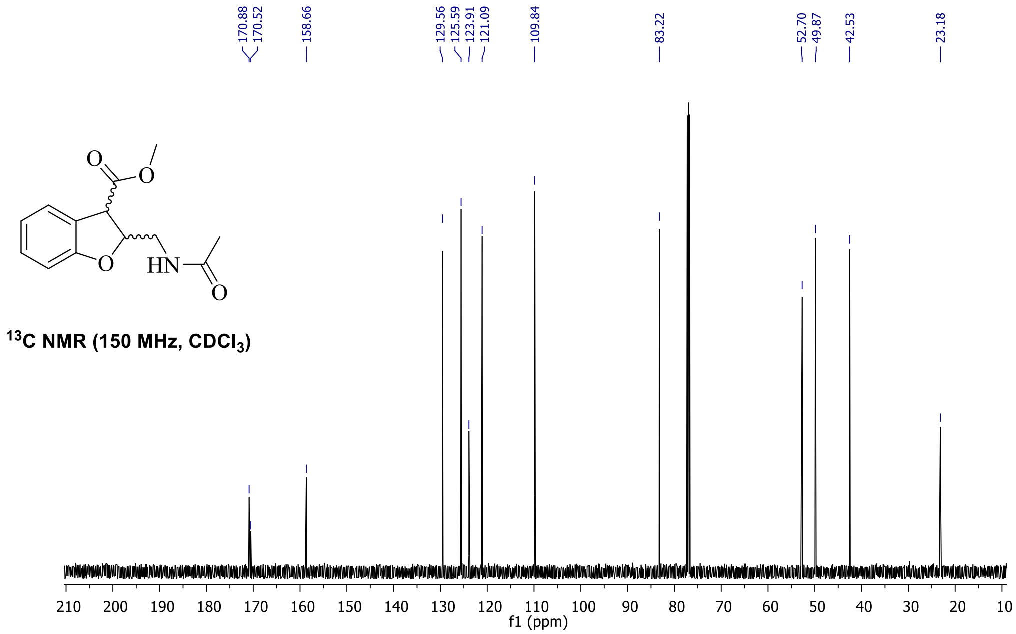

Figure S22: ^{13}C NMR spectrum of ***rac*-2a**

HRMS spectra

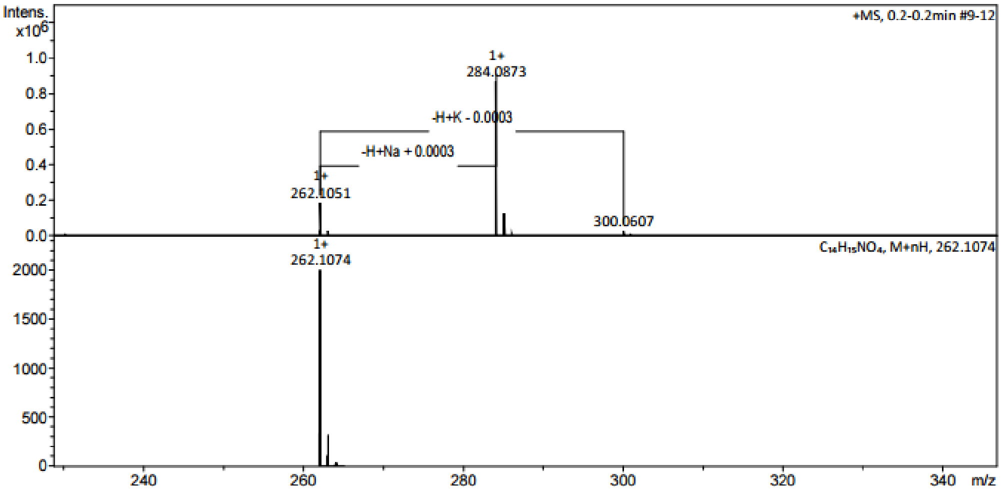

Figure S23: HRMS spectrum of **1**

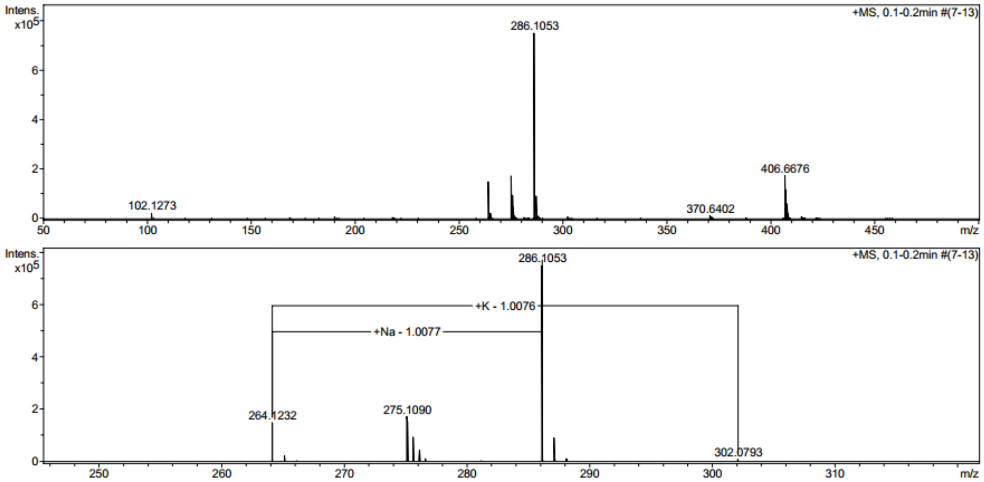

Figure S24: HRMS spectrum of ***rac*-1a**

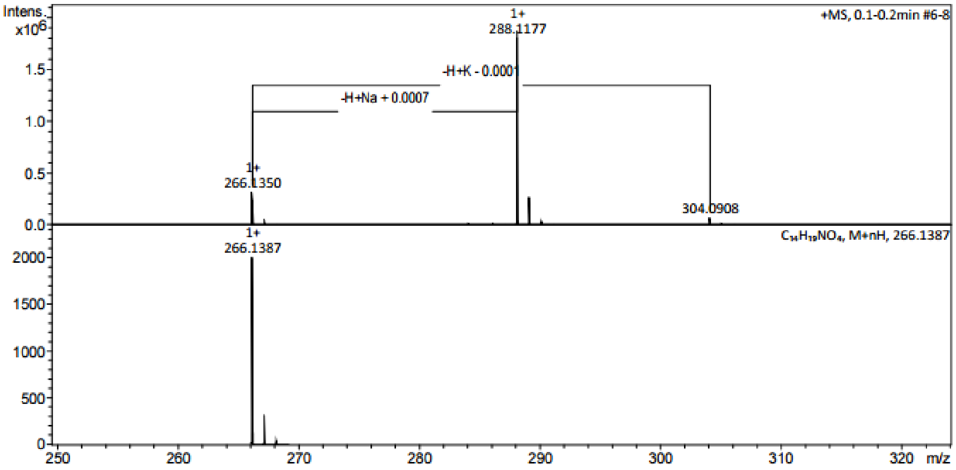

Figure S25: HRMS spectrum of **1b**

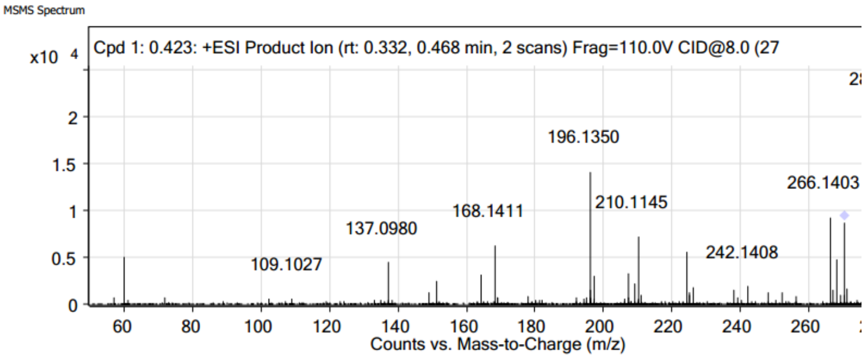

MS/MS Spectrum Peak List

m/z	z	Abund
60.0459		4964.82
168.1411	1	6207.09
196.135	1	14099.26
210.1145	1	7212.06
224.1299	1	5577.87
266.1403	1	9203.31
268.1561	1	4792.38
270.1721	1	8618.36

Figure S26: HRMS spectrum of **1c**

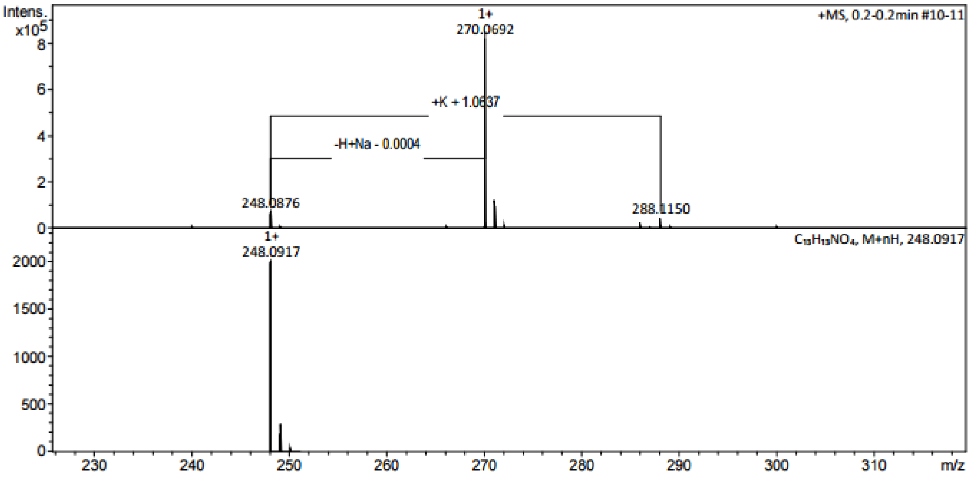

Figure S27: HRMS spectrum of **2**

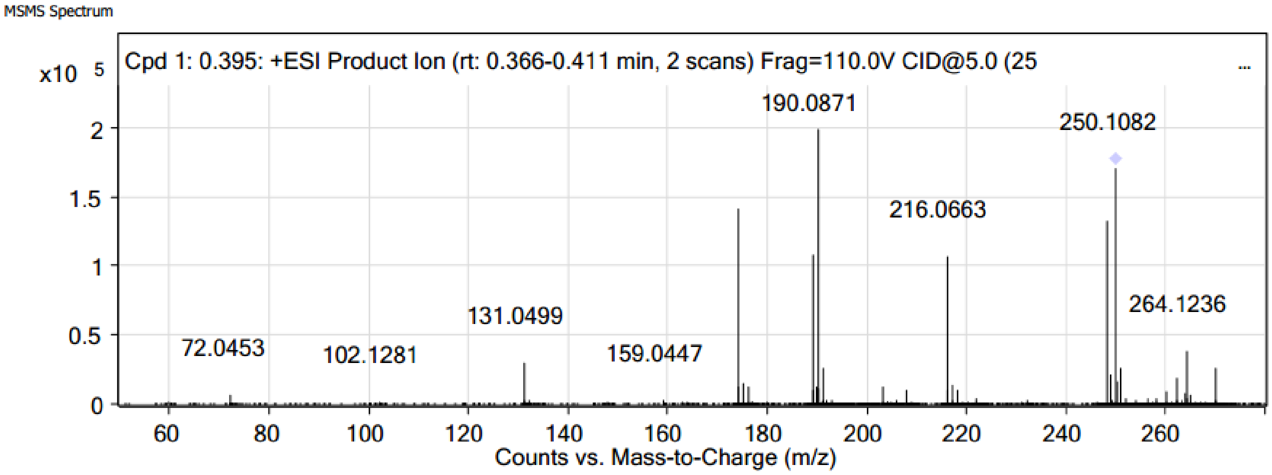

MS/MS Spectrum Peak List

m/z	z	Abund
131.0499	1	30081.78
174.0558	1	140817.73
189.0555	1	107451.94
190.0871	1	198593
191.0904	1	25845.61
216.0663	1	106361.93
248.0925	1	132246.02
250.1082	1	170271.02

Figure S28: HRMS spectrum of ***rac*-2a**

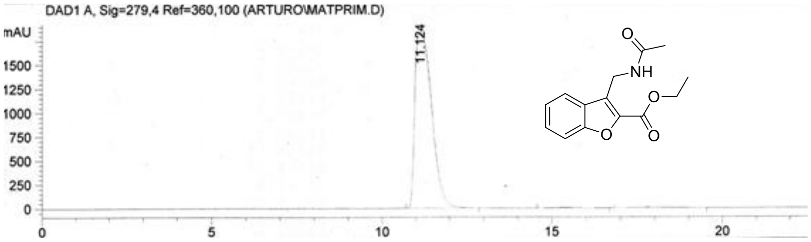

Figure S29: HPLC Data for **1**: Chiracel OJ-H (80:20 Hex:IPA, flow rate 0.6 mL/min, 279 nm), t_R= 11.12

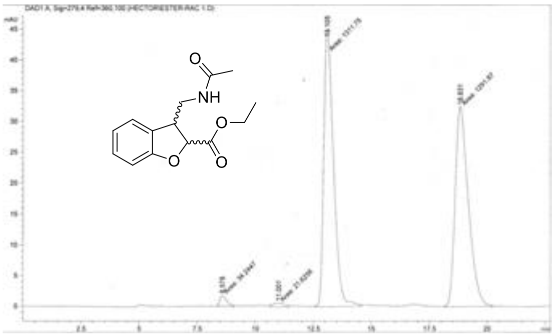

Figure S30: HPLC Data for ***rac*-1a**: Chiracel OJ-H (80:20 Hex:IPA, flow rate 0.6 mL/min, 279 nm), t_{R1}= 13.1 (A= 1311.75), t_{R2}= 18.83 (A= 1291.87).

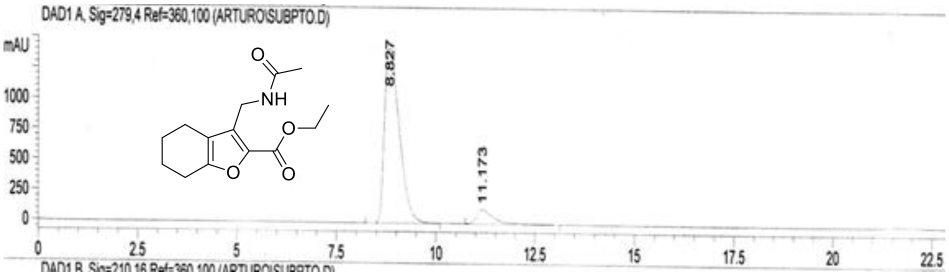

Figure S31: HPLC Data for **1b**: Chiracel OJ-H (80:20 Hex:IPA, flow rate 0.6 mL/min, 279 nm), t_R= 8.82

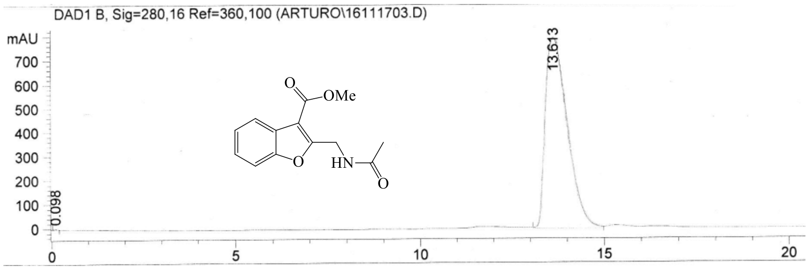

Figure S32: HPLC Data for **2**: Chiracel OJ-H (80:20 Hex:IPA, flow rate 0.6 mL/min, 279 nm), t_R= 13.61

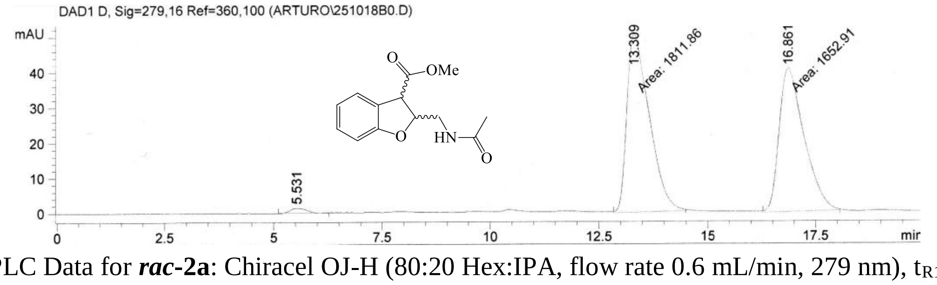

Figure S33: HPLC Data for ***rac*-2a**: Chiracel OJ-H (80:20 Hex:IPA, flow rate 0.6 mL/min, 279 nm), t_{R1}= 13.31 (A= 1811.8), t_{R2}= 16.86 (A= 1652.9).

Coordinates and Energies

Table S1. Compound 1 Neutral
B3LYP/6-31+G*
SCF Done: E(B3LYP) = -897.935548637 hartrees

Coordinates (from last standard orientation):

Center Number	Atomic Number	Atomic Type	Coordinates (Angstroms) X	Y	Z
1	6	0	-1.709737	-0.310672	-0.325331
2	6	0	-1.931348	-1.600576	0.201279
3	6	0	-2.810936	0.531158	-0.575553
4	6	0	-3.193789	-2.114005	0.481041
5	6	0	-4.084045	0.034712	-0.301436
6	6	0	-4.273643	-1.269124	0.213982
7	6	0	-0.277675	-0.167689	-0.480340
8	6	0	0.279929	-1.339294	-0.021489
9	8	0	-0.714510	-2.246702	0.394927
10	6	0	1.648376	-1.811013	0.131275
11	6	0	0.429147	1.036537	-1.045150
12	7	0	0.782217	2.025571	-0.020270
13	6	0	0.017211	3.136997	0.210992
14	6	0	0.486465	4.045613	1.329511
15	8	0	-1.013302	3.391882	-0.457957
16	8	0	1.998751	-2.955948	0.436726
17	8	0	2.562124	-0.788285	-0.095328
18	6	0	3.993741	-1.165920	-0.011686
19	6	0	4.799180	0.080732	-0.326064
20	1	0	-2.659325	1.544423	-0.931846
21	1	0	-3.321834	-3.110744	0.884000
22	1	0	-4.951201	0.662015	-0.478595
23	1	0	-5.281222	-1.618826	0.413368
24	1	0	1.341801	0.729027	-1.555740
25	1	0	-0.225311	1.544983	-1.756390
26	1	0	1.577096	1.817362	0.565833
27	1	0	0.527552	5.073830	0.959534
28	1	0	-0.245099	4.021155	2.144290
29	1	0	1.466979	3.769649	1.728409
30	1	0	4.186430	-1.550703	0.993898
31	1	0	4.176135	-1.975626	-0.724109
32	1	0	4.570377	0.453370	-1.329256
33	1	0	4.590564	0.876763	0.396057
34	1	0	5.869763	-0.147989	-0.280681

Table S2. Compound 1 Cation (N-1)
UB3LYP/6-31+G*
SCF Done: E(UB3LYP) = -897.640404403 hartrees

Coordinates (from last standard orientation):

Center Number	Atomic Number	Atomic Type	Coordinates (Angstroms) X	Y	Z
1	6	0	-1.709737	-0.310672	-0.325331
2	6	0	-1.931348	-1.600576	0.201279
3	6	0	-2.810936	0.531158	-0.575553
4	6	0	-3.193789	-2.114005	0.481041
5	6	0	-4.084045	0.034712	-0.301436
6	6	0	-4.273643	-1.269124	0.213982
7	6	0	-0.277675	-0.167689	-0.480340
8	6	0	0.279929	-1.339294	-0.021489
9	8	0	-0.714510	-2.246702	0.394927
10	6	0	1.648376	-1.811013	0.131275
11	6	0	0.429147	1.036537	-1.045150
12	7	0	0.782217	2.025571	-0.020270
13	6	0	0.017211	3.136997	0.210992
14	6	0	0.486465	4.045613	1.329511
15	8	0	-1.013302	3.391882	-0.457957
16	8	0	1.998751	-2.955948	0.436726
17	8	0	2.562124	-0.788285	-0.095328
18	6	0	3.993741	-1.165920	-0.011686
19	6	0	4.799180	0.080732	-0.326064
20	1	0	-2.659325	1.544423	-0.931846
21	1	0	-3.321834	-3.110744	0.884000
22	1	0	-4.951201	0.662015	-0.478595
23	1	0	-5.281222	-1.618826	0.413368
24	1	0	1.341801	0.729027	-1.555740
25	1	0	-0.225311	1.544983	-1.756390
26	1	0	1.577096	1.817362	0.565833
27	1	0	0.527552	5.073830	0.959534
28	1	0	-0.245099	4.021155	2.144290
29	1	0	1.466979	3.769649	1.728409
30	1	0	4.186430	-1.550703	0.993898
31	1	0	4.176135	-1.975626	-0.724109
32	1	0	4.570377	0.453370	-1.329256
33	1	0	4.590564	0.876763	0.396057
34	1	0	5.869763	-0.147989	-0.280681

Table S3. Compound 1 Anion (N+1)
UB3LYP/6-31+G*
SCF Done: E(UB3LYP) = -897.940537020 hartrees

Coordinates (from last standard orientation):

Center Number	Atomic Number	Atomic Type	Coordinates (Angstroms) X	Y	Z
1	6	0	-1.709737	-0.310672	-0.325331
2	6	0	-1.931348	-1.600576	0.201279
3	6	0	-2.810936	0.531158	-0.575553
4	6	0	-3.193789	-2.114005	0.481041
5	6	0	-4.084045	0.034712	-0.301436
6	6	0	-4.273643	-1.269124	0.213982
7	6	0	-0.277675	-0.167689	-0.480340
8	6	0	0.279929	-1.339294	-0.021489
9	8	0	-0.714510	-2.246702	0.394927
10	6	0	1.648376	-1.811013	0.131275
11	6	0	0.429147	1.036537	-1.045150
12	7	0	0.782217	2.025571	-0.020270
13	6	0	0.017211	3.136997	0.210992
14	6	0	0.486465	4.045613	1.329511
15	8	0	-1.013302	3.391882	-0.457957
16	8	0	1.998751	-2.955948	0.436726
17	8	0	2.562124	-0.788285	-0.095328
18	6	0	3.993741	-1.165920	-0.011686
19	6	0	4.799180	0.080732	-0.326064
20	1	0	-2.659325	1.544423	-0.931846
21	1	0	-3.321834	-3.110744	0.884000
22	1	0	-4.951201	0.662015	-0.478595
23	1	0	-5.281222	-1.618826	0.413368
24	1	0	1.341801	0.729027	-1.555740
25	1	0	-0.225311	1.544983	-1.756390
26	1	0	1.577096	1.817362	0.565833
27	1	0	0.527552	5.073830	0.959534
28	1	0	-0.245099	4.021155	2.144290
29	1	0	1.466979	3.769649	1.728409
30	1	0	4.186430	-1.550703	0.993898
31	1	0	4.176135	-1.975626	-0.724109
32	1	0	4.570377	0.453370	-1.329256
33	1	0	4.590564	0.876763	0.396057
34	1	0	5.869763	-0.147989	-0.280681

Table S4. Compound 2 Neutral
B3LYP/6-31+G*
SCF Done: E(B3LYP) = -858.629047373 hartrees

Coordinates (from last standard orientation):

Center Number	Atomic Number	Atomic Type	Coordinates (Angstroms) X	Y	Z
1	6	0	1.949714	0.127941	0.058945
2	6	0	1.956187	-1.244637	-0.242691
3	6	0	3.128195	0.724480	0.535247
4	6	0	3.061673	-2.072111	-0.100021
5	6	0	4.255820	-0.085810	0.688529
6	6	0	4.226150	-1.462084	0.377723
7	6	0	0.600906	0.596296	-0.232055
8	6	0	-0.121082	-0.487062	-0.686360
9	8	0	0.683729	-1.624922	-0.703289
10	6	0	-1.527302	-0.686492	-1.161480
11	7	0	-2.520835	-0.422047	-0.123201
12	6	0	-3.359314	-1.400297	0.347538
13	6	0	-4.313864	-0.969751	1.444598
14	8	0	-3.346041	-2.570271	-0.096154
15	6	0	0.162516	1.971287	-0.068175
16	8	0	-1.199835	2.150135	-0.300350
17	8	0	0.889114	2.924794	0.247974
18	6	0	-1.686063	3.538245	-0.226930
19	1	0	3.149745	1.781248	0.768431
20	1	0	3.016096	-3.125883	-0.344646
21	1	0	5.178625	0.351912	1.054656
22	1	0	5.122214	-2.058731	0.510757
23	1	0	-1.716533	-0.040158	-2.026551
24	1	0	-1.653769	-1.728389	-1.464034
25	1	0	-2.547928	0.520605	0.238084
26	1	0	-4.115290	-1.562183	2.343479
27	1	0	-4.234568	0.091770	1.696702
28	1	0	-5.339815	-1.186881	1.132375
29	1	0	-1.524455	3.940764	0.774790
30	1	0	-1.158468	4.157943	-0.954228
31	1	0	-2.746959	3.474394	-0.462062

Table S5. Compound 2 Cation (N-1)
UB3LYP/6-31+G*
SCF Done: E(UB3LYP) = -858.335277748 hartrees

Coordinates (from last standard orientation):

Center Number	Atomic Number	Atomic Type	Coordinates (Angstroms) X	Y	Z
1	6	0	1.949714	0.127941	0.058945
2	6	0	1.956187	-1.244637	-0.242691
3	6	0	3.128195	0.724480	0.535247
4	6	0	3.061673	-2.072111	-0.100021
5	6	0	4.255820	-0.085810	0.688529
6	6	0	4.226150	-1.462084	0.377723
7	6	0	0.600906	0.596296	-0.232055
8	6	0	-0.121082	-0.487062	-0.686360
9	8	0	0.683729	-1.624922	-0.703289
10	6	0	-1.527302	-0.686492	-1.161480
11	7	0	-2.520835	-0.422047	-0.123201
12	6	0	-3.359314	-1.400297	0.347538
13	6	0	-4.313864	-0.969751	1.444598
14	8	0	-3.346041	-2.570271	-0.096154
15	6	0	0.162516	1.971287	-0.068175
16	8	0	-1.199835	2.150135	-0.300350
17	8	0	0.889114	2.924794	0.247974
18	6	0	-1.686063	3.538245	-0.226930
19	1	0	3.149745	1.781248	0.768431
20	1	0	3.016096	-3.125883	-0.344646
21	1	0	5.178625	0.351912	1.054656
22	1	0	5.122214	-2.058731	0.510757
23	1	0	-1.716533	-0.040158	-2.026551
24	1	0	-1.653769	-1.728389	-1.464034
25	1	0	-2.547928	0.520605	0.238084
26	1	0	-4.115290	-1.562183	2.343479
27	1	0	-4.234568	0.091770	1.696702
28	1	0	-5.339815	-1.186881	1.132375
29	1	0	-1.524455	3.940764	0.774790
30	1	0	-1.158468	4.157943	-0.954228
31	1	0	-2.746959	3.474394	-0.462062

Table S6. Compound 2 Anion (N+1)
UB3LYP/6-31+G*
SCF Done: E(UB3LYP) = -858.631520281 hartrees

Coordinates (from last standard orientation):

Center Number	Atomic Number	Atomic Type	Coordinates (Angstroms) X	Y	Z
1	6	0	1.949714	0.127941	0.058945
2	6	0	1.956187	-1.244637	-0.242691
3	6	0	3.128195	0.724480	0.535247
4	6	0	3.061673	-2.072111	-0.100021
5	6	0	4.255820	-0.085810	0.688529
6	6	0	4.226150	-1.462084	0.377723
7	6	0	0.600906	0.596296	-0.232055
8	6	0	-0.121082	-0.487062	-0.686360
9	8	0	0.683729	-1.624922	-0.703289
10	6	0	-1.527302	-0.686492	-1.161480
11	7	0	-2.520835	-0.422047	-0.123201
12	6	0	-3.359314	-1.400297	0.347538
13	6	0	-4.313864	-0.969751	1.444598
14	8	0	-3.346041	-2.570271	-0.096154
15	6	0	0.162516	1.971287	-0.068175
16	8	0	-1.199835	2.150135	-0.300350
17	8	0	0.889114	2.924794	0.247974
18	6	0	-1.686063	3.538245	-0.226930
19	1	0	3.149745	1.781248	0.768431
20	1	0	3.016096	-3.125883	-0.344646
21	1	0	5.178625	0.351912	1.054656
22	1	0	5.122214	-2.058731	0.510757
23	1	0	-1.716533	-0.040158	-2.026551
24	1	0	-1.653769	-1.728389	-1.464034
25	1	0	-2.547928	0.520605	0.238084
26	1	0	-4.115290	-1.562183	2.343479
27	1	0	-4.234568	0.091770	1.696702
28	1	0	-5.339815	-1.186881	1.132375
29	1	0	-1.524455	3.940764	0.774790
30	1	0	-1.158468	4.157943	-0.954228
31	1	0	-2.746959	3.474394	-0.462062

Table S7. Energies and atomic charges in the frame B3LYP/6-31+G* of the molecules 1

	MOLECULE 1											
	N		N+1		N-1		f+	f-	Local softness		global softness
	# e-	CHARGE	# e-	CHARGE	# e-	CHARGE	(N+1)-(N)	(N)-(N-1)	$s_x^+ = f_x^+ s$	$s_x^- = f_x^- s$	**0.1267**
C1	6.021074757	-0.021074757	6.026982508	-0.026982507	6.007236492	-0.007236	0.005908	0.013838265	0.0007	0.0018	0.1267
C2	5.667178459	0.33282154	5.673981369	0.32601863	5.666571274	0.333428725	0.00680291	0.000607185	0.0009	0.0001	0.1267
C3	6.004332399	-0.004332399	6.049416645	-0.049416645	5.962146437	0.037853562	0.045084246	0.042185962	0.0057	0.0053	0.1267
C4	5.991481981	0.008518019	6.028808744	-0.028809	5.943214962	0.056785037	0.037326764	0.048267	0.0047	0.0061	0.1267
C5	6.018868941	-0.018869	6.044342949	-0.044342949	5.987886043	0.012113956	0.025474008	0.030982898	0.0032	0.0039	0.1267
C6	6.009590366	-0.009590366	6.055767147	-0.055767147	5.967971088	0.032028911	0.046177	0.041619278	0.0059	0.0053	0.1267
C7	5.976263598	0.023736402	6.040871631	-0.040871631	5.947151777	0.052848222	0.064608033	0.029111821	0.0082	0.0037	0.1267
C8	5.681799014	0.318200985	5.716844354	0.283155646	5.62674896	0.373251039	0.03504534	0.055050	0.0044	0.0070	0.1267
O9	8.832477042	-0.832477042	8.874083244	-0.874083244	8.766030852	-0.766030852	0.041606202	0.06644619	0.0053	0.0084	0.1267
C10	4.801285262	1.198714737	4.897687749	1.102312251	4.781261055	1.218738945	0.096402487	0.020024207	0.0122	0.0025	0.1267
C11	5.647104465	0.352895534	5.652089925	0.347910074	5.651514193	0.348485806	0.004985	-0.004409728	0.0006	-0.0006	0.1267
N12	7.993509274	-0.993509274	7.987434776	-0.987434776	7.964259423	-0.964259423	-0.006074498	0.029249851	-0.0008	0.0037	0.1267
C13	4.870980013	1.129019986	4.874167304	1.125832696	4.891312373	1.108687627	0.003187	-0.02033236	0.0004	-0.0026	0.1267
C14	6.001097439	-0.001097439	6.00208992	-0.00208992	5.997253588	0.002746411	0.000992481	0.003843851	0.0001	0.0005	0.1267
O15	8.995021654	-0.995021654	9.012467433	-1.012467433	8.907926801	-0.907926801	0.017445779	0.087094853	0.0022	0.0110	0.1267
O16	8.937119596	-0.937119596	9.055223193	-1.055223193	8.860032219	-0.860032219	0.118104	0.077087	0.0150	0.0098	0.1267
O17	8.875444102	-0.875444102	8.912800948	-0.912800948	8.861591055	-0.861591055	0.037356846	0.013853047	0.0047	0.0018	0.1267
C18	5.641334078	0.358665921	5.612968872	0.387031127	5.661388661	0.338611338	-0.028365206	-0.020054583	-0.0036	-0.0025	0.1267
C19	5.975327627	0.024672372	5.978053034	0.021946965	5.974966569	0.02503343	0.002725407	0.000361058	0.0003	0.0000	0.1267
H20	0.909361548	0.090638451	0.954075527	0.045924472	0.866027483	0.133972516	0.044713979	0.043334065	0.0057	0.0055	0.1267
H21	0.938606566	0.061393433	0.989108153	0.010891846	0.877239573	0.122760426	0.050501587	0.061366993	0.0064	0.0078	0.1267
H22	0.966971716	0.033028283	1.020826873	-0.020826873	0.908775467	0.091224532	0.053855157	0.058196249	0.0068	0.0074	0.1267
H23	0.965974469	0.03402553	1.025647444	-0.02564744	0.900663539	0.09933646	0.059673	0.06531093	0.0076	0.0083	0.1267
H24	0.945770424	0.054229575	0.970073247	0.029926752	0.908471038	0.091528961	0.024302823	0.037299386	0.0031	0.0047	0.1267
H25	0.929891914	0.070108085	0.961948346	0.038051653	0.908404976	0.091595023	0.032056432	0.021486938	0.0041	0.0027	0.1267
H26	0.616078574	0.383921426	0.62763677	0.372363229	0.585483547	0.414516452	0.011558196	0.030595	0.0015	0.0039	0.1267
H27	0.954461938	0.045538061	0.981565088	0.018434911	0.918719626	0.081280373	0.02710315	0.035742	0.0034	0.0045	0.1267
H28	0.957265467	0.042734532	0.973839484	0.026160515	0.931543061	0.068456938	0.016574017	0.025722	0.0021	0.0033	0.1267
H29	0.995472988	0.004527011	1.006894272	-0.006894272	0.968171555	0.031828444	0.011421	0.027301	0.0014	0.0035	0.1267
H30	0.957428179	0.04257182	0.982396449	0.017603551	0.938450211	0.061549788	0.02496827	0.018977968	0.0032	0.0024	0.1267
H31	0.956754249	0.04324575	0.981125838	0.018874161	0.936857919	0.06314208	0.024371589	0.01989633	0.0031	0.0025	0.1267
H32	0.98195806	0.018041939	0.995431024	0.004568975	0.975866489	0.02413351	0.013472964	0.006091571	0.0017	0.0008	0.1267
H33	0.990584716	0.009415285	1.004652316	-0.004652316	0.986559961	0.013440038	0.0140676	0.004025	0.0018	0.0005	0.1267
H34	0.982700847	0.017299152	1.019734333	-0.019734333	0.953306273	0.046693726	0.037033486	0.029394574	0.0047	0.0037	0.1267

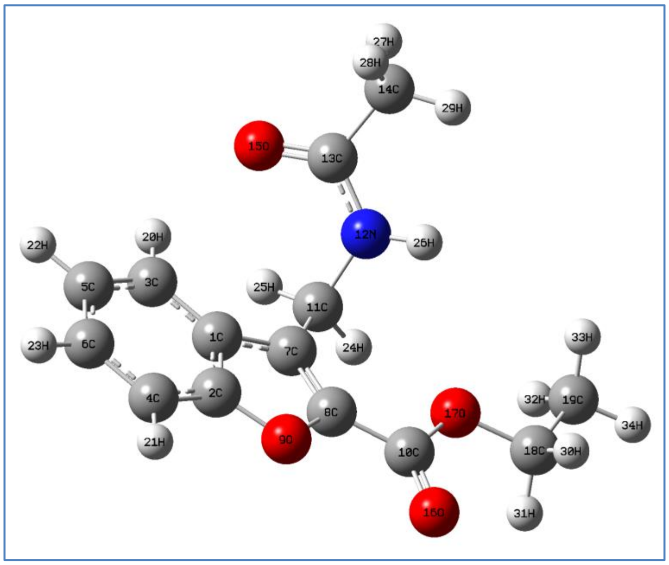

Figure S34: Optimized geometry of **1**

Table S8. Energies and atomic charges in the frame B3LYP/6-31+G* of the molecules 2

	MOLECULE 2										
	N		N+1		N-1		*ƒ*+	*ƒ*-	Local softness		global softness
	# e-	CHARGE	# e-	CHARGE	# e-	CHARGE	(N+1)-(N)	(N)-(N-1)	$s_x^+ = f_x^+ s$	$s_x^- = f_x^- s$	**0.126**
C1	6.000466272	-0.000466272	6.003740313	-0.003740313	5.98349261	0.016507	0.003274	0.016973662	0.0004	0.0021	0.126
C2	5.697576887	0.302423112	5.686883916	0.313116083	5.68476851	0.315231489	-0.010692971	0.012808377	-0.0013	0.0016	0.126
C3	5.994254446	0.005745553	6.027000896	-0.027000896	5.954797986	0.045202013	0.03274645	0.03945646	0.0041	0.0050	0.126
C4	5.987415531	0.012584468	6.027931146	-0.027931	5.947360146	0.052639853	0.040515615	0.040055	0.0051	0.0050	0.126
C5	6.017567565	-0.017568	6.047732016	-0.047732016	5.990199961	0.009800038	0.030164451	0.027367604	0.0038	0.0034	0.126
C6	6.010504411	-0.010504411	6.047190871	-0.047190871	5.966291724	0.033708275	0.036686	0.044212687	0.0046	0.0056	0.126
C7	6.00002032	-0.00002032	6.027055211	-0.027055211	5.965911086	0.034088913	0.027034891	0.034109234	0.0034	0.0043	0.126
C8	5.63161525	0.368384749	5.725108558	0.274891441	5.595873071	0.404126928	0.093493308	0.035742	0.0118	0.0045	0.126
O9	8.844857816	-0.844857816	8.89981567	-0.89981567	8.803040681	-0.803040681	0.054957854	0.041817135	0.0069	0.0053	0.126
C10	5.638232079	0.36176792	5.639390141	0.360609859	5.641018357	0.358981642	0.001158062	-0.002786278	0.0001	-0.0004	0.126
N11	8.004243655	-1.004243655	7.997814262	-0.997814262	7.969921882	-0.969921882	-0.006429	0.034321773	-0.0008	0.0043	0.126
C12	4.865851448	1.134148551	4.873323838	1.126676161	4.899546378	1.100453622	0.00747239	-0.03369493	0.0009	-0.0042	0.126
C13	6.000781075	-0.000781075	6.001756884	-0.001756884	5.993136547	0.006863452	0.000976	0.007644528	0.0001	0.0010	0.126
O14	8.984215202	-0.984215202	9.020920934	-1.020920934	8.836723105	-0.836723105	0.036705732	0.147492097	0.0046	0.0186	0.126
C15	4.83078489	1.169215109	4.925311433	1.074688566	4.811040208	1.188959791	0.094526543	0.019744682	0.0119	0.0025	0.126
O16	8.879603905	-0.879603905	8.910990199	-0.910990199	8.872847672	-0.872847672	0.031386	0.006756	0.0040	0.0009	0.126
O17	8.963950907	-0.963950907	9.076941385	-1.076941385	8.904512209	-0.904512209	0.112990478	0.059438698	0.0142	0.0075	0.126
C18	5.650815184	0.349184815	5.62947789	0.370522109	5.665215297	0.334784702	-0.021337294	-0.014400113	-0.0027	-0.0018	0.126
H19	0.92358235	0.076417649	0.951575196	0.048424803	0.872870301	0.127129698	0.027992846	0.050712049	0.0035	0.0064	0.126
H20	0.937922924	0.062077075	0.986685398	0.013314601	0.882625954	0.117374045	0.048762474	0.05529697	0.0061	0.0070	0.126
H21	0.968384439	0.03161556	1.019487153	-0.019487153	0.911729328	0.088270671	0.051102714	0.056655111	0.0064	0.0071	0.126
H22	0.965786216	0.034213783	1.020214242	-0.020214242	0.901760828	0.098239171	0.054428026	0.064025388	0.0069	0.0081	0.126
H23	0.954256189	0.04574381	1.001821449	-0.001821449	0.913274519	0.08672548	0.047565	0.04098167	0.0060	0.0052	0.126
H24	0.889780308	0.110219691	0.9299909	0.070009099	0.872200167	0.127799832	0.040210592	0.017580141	0.0051	0.0022	0.126
H25	0.590921933	0.409078066	0.593758127	0.406241872	0.563223831	0.436776168	0.002836194	0.027698102	0.0004	0.0035	0.126
H26	0.957068741	0.042931258	0.980106515	0.019893484	0.922946947	0.077053052	0.023037774	0.034122	0.0029	0.0043	0.126
H27	0.99763743	0.002362569	1.003777767	-0.003777767	0.964676053	0.035323946	0.006140337	0.032961	0.0008	0.0042	0.126
H28	0.958025841	0.041974158	0.987230894	0.012769105	0.917622856	0.082377143	0.029205053	0.040403	0.0037	0.0051	0.126
H29	0.945281824	0.054718175	0.975548377	0.024451622	0.924976574	0.075023425	0.030267	0.020305	0.0038	0.0026	0.126
H30	0.943674844	0.056325155	0.973530021	0.026469978	0.921501876	0.078498123	0.029855177	0.022172968	0.0038	0.0028	0.126
H31	0.95686339	0.043136609	0.999459033	0.000540966	0.934863678	0.065136321	0.042595643	0.021999712	0.0054	0.0028	0.126

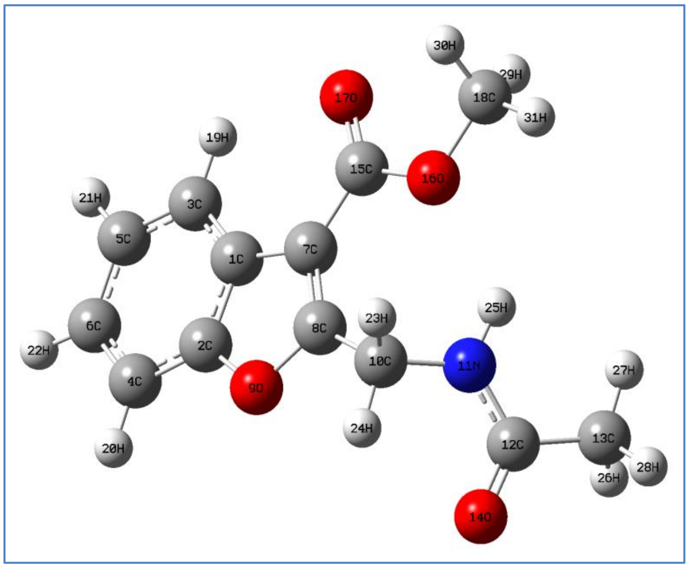

Figure S35: Optimized geometry of **2**
